# Conversational Chatbot for Cigarette Smoking Cessation: Results From the 11-Step User-Centered Design Development Process and Randomized Controlled Trial

**DOI:** 10.2196/57318

**Published:** 2024-07-23

**Authors:** Jonathan B Bricker, Brianna Sullivan, Kristin Mull, Margarita Santiago-Torres, Juan M Lavista Ferres

**Affiliations:** 1 Division of Public Health Sciences Fred Hutch Cancer Center Seattle, WA United States; 2 Department of Psychology University of Washington Seattle, WA United States; 3 AI for Good Lab Microsoft Corporation Redmond, WA United States

**Keywords:** chatbot, conversational agent, conversational agents, digital therapeutics, smoking cessation, development, develop, design, smoking, smoke, smokers, quit, quitting, cessation, chatbots, large language model, LLM, LLMs, large language models, addict, addiction, addictions, mobile phone

## Abstract

**Background:**

Conversational chatbots are an emerging digital intervention for smoking cessation. No studies have reported on the entire development process of a cessation chatbot.

**Objective:**

We aim to report results of the user-centered design development process and randomized controlled trial for a novel and comprehensive quit smoking conversational chatbot called *QuitBot*.

**Methods:**

The 4 years of formative research for developing QuitBot followed an 11-step process: (1) specifying a conceptual model; (2) conducting content analysis of existing interventions (63 hours of intervention transcripts); (3) assessing user needs; (4) developing the chat’s persona (“personality”); (5) prototyping content and persona; (6) developing full functionality; (7) programming the QuitBot; (8) conducting a diary study; (9) conducting a pilot randomized controlled trial (RCT); (10) reviewing results of the RCT; and (11) adding a free-form question and answer (QnA) function, based on user feedback from pilot RCT results. The process of adding a QnA function itself involved a three-step process: (1) generating QnA pairs, (2) fine-tuning large language models (LLMs) on QnA pairs, and (3) evaluating the LLM outputs.

**Results:**

We developed a quit smoking program spanning 42 days of 2- to 3-minute conversations covering topics ranging from motivations to quit, setting a quit date, choosing Food and Drug Administration–approved cessation medications, coping with triggers, and recovering from lapses and relapses. In a pilot RCT with 96% three-month outcome data retention, QuitBot demonstrated high user engagement and promising cessation rates compared to the National Cancer Institute’s SmokefreeTXT text messaging program, particularly among those who viewed all 42 days of program content: 30-day, complete-case, point prevalence abstinence rates at 3-month follow-up were 63% (39/62) for QuitBot versus 38.5% (45/117) for SmokefreeTXT (odds ratio 2.58, 95% CI 1.34-4.99; *P*=.005). However, Facebook Messenger intermittently blocked participants’ access to QuitBot, so we transitioned from Facebook Messenger to a stand-alone smartphone app as the communication channel. Participants’ frustration with QuitBot’s inability to answer their open-ended questions led to us develop a core conversational feature, enabling users to ask open-ended questions about quitting cigarette smoking and for the QuitBot to respond with accurate and professional answers. To support this functionality, we developed a library of 11,000 QnA pairs on topics associated with quitting cigarette smoking. Model testing results showed that Microsoft’s Azure-based QnA maker effectively handled questions that matched our library of 11,000 QnA pairs. A fine-tuned, contextualized GPT-3.5 (OpenAI) responds to questions that are not within our library of QnA pairs.

**Conclusions:**

The development process yielded the first LLM-based quit smoking program delivered as a conversational chatbot. Iterative testing led to significant enhancements, including improvements to the delivery channel. A pivotal addition was the inclusion of a core LLM–supported conversational feature allowing users to ask open-ended questions.

**Trial Registration:**

ClinicalTrials.gov NCT03585231; https://clinicaltrials.gov/study/NCT03585231

## Introduction

### Background

Cigarette smoking accounts for 8 million premature deaths and 25% of all cancer deaths annually [1,2]. Despite advancements in government policies, antismoking campaigns, and shifting societal norms, existing smoking cessation interventions continue to have limited treatment engagement and cessation rates [3-9]. While this is a problem for the general population of people who smoke, the issue is particularly pronounced in communities considered marginalized, synonymous with groups considered vulnerable or disadvantaged, which are segments of society facing systemic disadvantages and barriers in accessing resources and opportunities. Populations considered marginalized, marked by factors such as racial or ethnic minority status, sexual or gender identity differences, low education and income levels, higher unemployment rates, or an increased prevalence of mental illness, encounter discrimination, social exclusion, and limited influence in decision-making processes.

Challenges in treatment engagement and cessation efficacy across all communities of people who smoke are compounded by a scarcity of trained clinicians and significant barriers, including cost and lack of insurance, hindering access to existing clinician-delivered interventions [10-14]. Given that 1.3 billion people in the world smoke cigarettes, with 70% of them wanting to quit, it would be impractical to have enough trained clinicians to help people quit smoking [15,16]. Indeed, only 5% of cessation attempts are aided by a health professional [17]. Consequently, there is an enormous need for high-impact, cost-effective, population-level interventions for smoking cessation.

A well-documented finding from research on clinician-delivered treatments has emphasized the significance of therapeutic conversations as powerful drivers of patient engagement [18-21]. Therapeutic conversations, which form a social-emotional bond with the user, have predicted treatment engagement and, subsequently, health outcomes across various treatments and settings [12,18,22]. A new technology provides an opportunity to leverage engaging therapeutic conversations. Advances in machine learning, large language models (LLMs), and cloud computing are now making it possible to create and widely disseminate conversational chatbots for behavior change coaching.

Unlike the chatbots used in customer service contexts, conversational chatbots for behavior change coaching are designed to form long-term social-emotional connections with users, even as they are made aware that chatbots are merely computer software that use language to communicate with users [23,24]. Conversational chatbots for coaching are designed to be supportive and empathic, offering reflective listening, personalized responses, and timely advice aligned with the user’s individual needs [25,26]. In the context of cessation, conversational chatbots can enhance engagement through an informal therapeutic conversational style tailored to users’ unique barriers to quitting smoking [27]. Conversational chatbots only require a text response to operate, making them ideal for all individuals who smoke, including those with low technology literacy [26]. Overall, conversational chatbots offer a cost-effective communication platform, accessible at any time, and have the potential for high population-level reach, making them a valuable tool in smoking cessation interventions.

To date, research on conversational chatbots for smoking cessation is scarce. Existing literature revealed a limited number of empirical studies, often exhibiting low methodological quality [28]. There is a notable paucity of randomized controlled trials (RCTs) focusing on conversational chatbots for smoking cessation, and while promising results have emerged, they have been limited by low quit rates [29]. Several conversational chatbots for smoking cessation in the public domain include Florence (World Health Organization) [30], Bella (Solutions4Health) [31], and Alex AI (Alex Therapeutics) [32]. However, we are not aware of publications on their efficacy, with only the Florence app having reported user’s receptivity results [33]. Critical to creating useful and engaging conversational chatbots is following a user-centered design development process [34]. Similar to most chatbots, the development of the chatbots listed above lacks context for how they were designed and any user-centered design that involved conducting a needs assessment or including user feedback during the development process [28,35]. The few studies that have provided development details only describe early design phases, such as coding 30 quit coaching calls for prototype development, without empirical efficacy data [28,35,36]. In sum, the literature on chatbots for smoking cessation offers only partial accounts on how they were developed or report on initial stages of development.

### Objective

To address these gaps, this paper describes the comprehensive 4-year, 11-step user-centered design development process for a novel quit smoking conversational chatbot named “QuitBot.” This single report aims to summarize the entirety of the QuitBot development process.

## Methods

### Overview of the Formative Research Process

**Figure 1:**
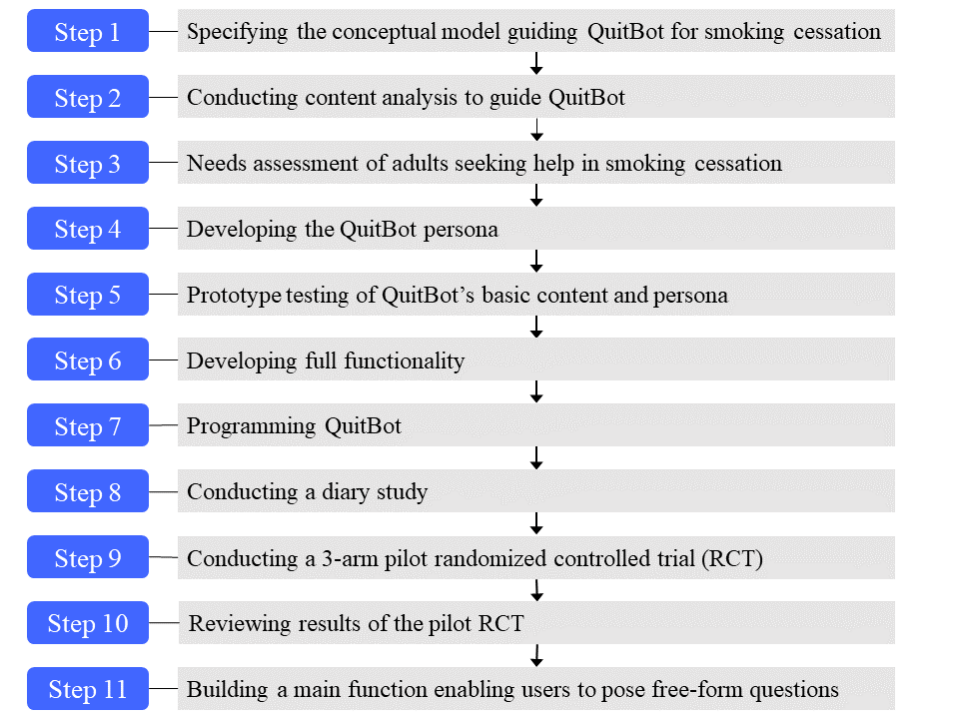
Overview of QuitBot’s formative research process.

The 4 years of formative research for developing QuitBot followed an 11-step process, consistent with a user-centered design framework (Figure 1) [37,38].

The steps were as follows: (1) specifying a conceptual model to guide the QuitBot intervention targets; (2) conducting content analysis of existing smoking cessation interventions to guide the QuitBot coaching conversations; (3) conducting a needs assessment to determine what an adult seeking help in quitting smoking would need from a cessation chatbot; (4) developing the QuitBot persona, or personality of the chatbot, to shape the user’s experience of and bond with the QuitBot chatbot; (5) prototyping QuitBot’s basic content and persona; (6) developing the full functionality of the QuitBot; (7) programming the QuitBot; (8) conducting a diary study for user feedback on their interactions with QuitBot and its design and content; (9) conducting a pilot RCT to test QuitBot for smoking cessation; (10) reviewing results of the pilot RCT; and (11) adding a free-form question and answer (QnA) function, based on user feedback from pilot RCT results. The process of adding the QnA function itself involved a three-step process: (1) generating QnA pairs, (2) fine-tuning LLMs on the QnA pairs, and (3) evaluating the LLM model outputs.

### Step 1: Specifying the Conceptual Model Guiding QuitBot for Smoking Cessation

**Figure 2:**
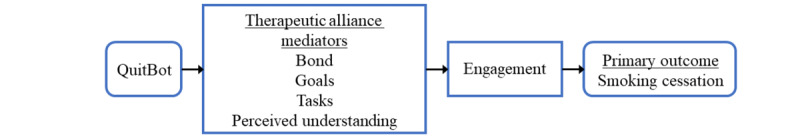
Conceptual model of QuitBot for smoking cessation.

The conceptual model guiding the development of QuitBot for smoking cessation (Figure 2) focuses on impacting user engagement through 4 therapeutic alliance processes. The four processes are as follows: (1) *bond* with QuitBot, (2) agreement on smoking cessation *goal,* (3) agreement on *tasks* for achieving smoking cessation goal, and (4) *perception that QuitBot understands* user’s current needs [18].

These working alliance processes have predicted smoking cessation [39] and quit attempts [40] and have mediated the impact of human therapist–delivered smoking cessation interventions [40]. QuitBot uses various strategies to establish a therapeutic alliance, including expressing empathy for the user’s struggles [41,42], engaging in social dialogue [43,44], using metarelational communication (ie, discuss the relationship) [45], and expressing happiness while interacting with the user [42]. Language constructs such as inclusive pronouns [41], politeness strategies [46], and the use of greetings and farewells rituals [47] contribute to the creation of this alliance as well. Compared to a technology that did not use these verbal behaviors, a conversational chatbot for physical activity increased these therapeutic alliance processes, which in turn was predictive of higher engagement with the chatbot [48].

Agreement on smoking cessation goal starts by collaboratively setting a quit date (eg, “Have you thought about a specific day you would like to quit? Generally, I recommend about 14 days away.”). QuitBot enhances perceived understanding by promptly addressing the user’s immediate needs (eg, “You say you are tempted by friends who smoke. Here’s a tip that might help...”). In addition, self-disclosure [49] is used to foster perceived understanding, generating various positive outcomes, especially when the listener responds with support and validation [50]. A chatbot that used self-disclosure increased the user’s perception that the chatbot understood their needs, which in turn predicted more positive mood [51].

### Step 2: Conducting Content Analysis to Guide QuitBot

The content analysis aimed to establish a natural flow of coaching conversations for QuitBot, aligned with US Clinical Practice Guidelines for smoking cessation [52]. In the initial phase of the content analysis, we interviewed a panel of experts, including 3 smoking cessation counselors, a smoking cessation master trainer, and a tobacco cessation scientist from our team. This panel consisted of 4 women and 1 man, with 20% (1/5) from racial and ethnic minority backgrounds. Among them, 40% (2/5) held a PhD in clinical psychology, while 60% (3/5) had master’s degrees in counseling or social work. Collectively, they had a wealth of experience ranging from 3 to 20 years, with an average of 8 (SD 4.6) years, in developing and delivering smoking cessation interventions. Deductive coding of these interviews and expert consensus iteratively lead to the formulation of common themes, domain-specific responses, and anticipated user interactions that QuitBot should address. We identified common conversation topics about smoking cessation, including triggers to smoke (ie, physical, emotional, and situational triggers), motivations to quit, and barriers to quitting. Interviews also highlighted the importance of QuitBot’s persona to be sensitive and empathetic to the user and to express that their concerns are being heard.

Guided by this expert consensus on conversation topics, the second phase was to extract the content and flow of smoking interventions as they naturally occur in actual interactions between cessation counselors and patients. To achieve this, we conducted semantic analysis of verbatim manually transcribed intervention conversation transcripts obtained from our telephone counseling intervention trial, randomly selected among those who did and did not quit smoking (R01 DA038411) [53]. A total of 159 call transcripts (equating to 63 h and 23 min) from 117 unique participants were randomly selected, constituting a 7.8% (159/2038) sample from each of the 5 sessions (with an average session duration of 22.9 min) of an efficacious behavioral intervention for smoking cessation with a 25% thirty-day point prevalence abstinence (PPA) rate at the 12-month follow-up [53]. These sessions covered various topics, including motivations to quit, triggers to smoke, barriers to quitting, setting a quit date, developing a quit plan, education and proper use of Food and Drug Administration (FDA)–approved medications for quitting smoking, coping skills for dealing with urges, enlisting social support, and strategies for avoiding external cues to smoke. Participants had a mean age of 47.4 (SD 12.7) years, with 43.6% (51/117) male participants and 21.4% (25/117) from racial and ethnic minority backgrounds.

Transcripts underwent deductive coding using a predefined codebook to identify common conversation topics related to smoking cessation, such as triggers to smoke (ie, physical, emotional, and situational triggers), motivations to quit, and barriers to quitting. Using a supervised machine learning approach, these topics formed the basis of QuitBot’s entity extraction, wherein elements of the unstructured transcript text were coded into predefined categories. Subsequently, we determined intent classifications, which involved discerning the meaning of the user’s text. Finally, we mapped out the natural conversational flow of both the chatbot and the range of verbal responses and comments that users might provide in response to the chatbot. The entity extraction, intent classifications, and conversational mapping were conducted using the LUIS conversational artificial intelligence (AI) program [54].

### Step 3: Needs Assessment of Adults Seeking Help in Smoking Cessation

Assessing the needs of adults seeking help in smoking cessation interventions shapes what the user should be able to do with a chatbot. To assess user needs, we first analyzed the results of the content analysis phase. Subsequently, we conducted interviews with 5 adults who had participated in our human-delivered smoking cessation interventions within the past year (including 2 who quit and 3 who did not quit) [55]. Participants had a mean age of 46.1 (SD 10.4) years, with 40% (2/5) female participants and 40% (2/5) from racial and ethnic minority backgrounds. The interviews queried participants about their personal background and smoking history, expectations for a smoking cessation program, experiences with a human cessation coach, perceptions regarding setting, keeping and changing quit dates, coping skills for urges to smoke, and attitudes toward and expectations of what a chatbot could do for helping them quit smoking. Semistructured interviews were conducted in person at the lead author’s user experience (UX) Research HABIT laboratory. The deductive thematic analysis organized the user’s responses by grouping them into themes, reviewing the themes, and then interpreting them [56-58]. The themes of the key user needs identified were (1) a coach who can make a personal connection, (2) on-demand help with urges, and (3) skills for preparation to quit and preventing relapse.

### Step 4: Developing the QuitBot “Persona”

The user’s bond with the chatbot is impacted by its persona [48]. On the basis of interviews with smoking cessation coaches and our master trainer, we created the persona to foster a strong bond with users. Presented to the user as a computer program (eg, “I’m a bot designed to help you live smoke free”), elements of the QuitBot persona included expressions of empathy [41,42], social dialogue [43,44], metarelational communication (ie, discuss the relationship) [45], and expressing happiness to see the user [42]. In addition, specific language constructs, including inclusive pronouns [41], politeness strategies [46], and greetings and farewells rituals, were integrated to enhance the UX and promote a respectful dialogue [47]. Finally, we established 11 core values for the persona, serve as guiding principles for QuitBot’s behavior throughout conversations.

### Step 5: Prototype Testing of QuitBot’s Basic Content and Persona

The prototyping testing of QuitBot’s basic content and persona aimed to assess users’ initial responses to basic smoking cessation conversations between the user and the persona. Stimuli were built using botmock [59,60] to develop the chat dialogue, which was then integrated into Facebook Messenger (FM; Meta Platforms, Inc) using Chatfuel [61]. Participants had a guided initial chat conversation introducing the chatbot and program goals, querying about triggers for smoking, and setting a quit date. Subsequently, they interacted with QuitBot for a second conversation, focusing on tracking triggers to smoke. For both conversations, a UX researcher frequently paused to prompt participants to think-aloud their experiences with QuitBot. Real-time interactions between the user and QuitBot were facilitated by a UX researcher using the Chatfuel program [61]. To evaluate this process, 75-minute individual interviews were conducted with 8 adults interested in quitting smoking. Four were chosen because they thought a chatbot could be helpful for quitting smoking, while the remaining 4 were selected because they were unsure or skeptical that a chatbot would be helpful for quitting smoking. The mean age of the participants was 42 (SD 11.1) years, with 38% (3/8) male participants, 50% (4/8) female participants, and 12% (1/8) transgender participants. In addition, 38% (3/8) of the participants had high school education or less and 25% (2/8) reported being from racial or ethnic minority backgrounds.

### Semistructured Interviews

Semistructured interviews were conducted in person at the lead author’s UX Research HABIT laboratory. A deductive thematic analysis method was used to organize user responses into themes, review those themes, and then interpret them [56-58]. Despite initial skepticism from half of participants (4/8, 50%) regarding the usefulness of interacting with a digital coach, the results showed a notable shift in the interest in QuitBot by the end of the interview: 100% (8/8) reported that a chatbot such as QuitBot would be valuable for helping someone quit smoking, with 88% (7/8) expressing willingness to try this chatbot for quitting. In addition, all participants (8/8, 100%) found QuitBot easy to use, noting its conversational tone as “encouraging,” “polite,” and “reassuring.” They deemed the length and speed of onboarding conversations appropriate and felt comfortable providing conversational responses. Participants expressed surprise at the “humanness” of QuitBot’s avatar, noting its informal, reassuring, accessible, and easy-to-talk-to demeanor.

When discussing whether the avatar should have a gender or a name, there was consensus among participants in favor of a female persona, with the name “Ellen” deemed appropriate (interestingly, one of the initial participants spontaneously suggested “How about a woman named ‘Ellen’?”). Later participants concurred with this choice when asked by the UX researcher.

Participants also expressed a desire for more actionable suggestions and to open and close each conversation with a specific plan of action. In response, we added a plan outlining what to anticipate, letting them know that the avatar would initiate a chat the following day and introduce a new quitting smoking skill in the subsequent conversation. Some participants wanted additional time to decide on a quit date, prompting us to include a dialogue indicating that they postponed setting a quit date until they felt ready. In addition, participants suggested visualizing their progress in quitting smoking, such as through a graph. In response, we added a progress chart displaying the number of cigarettes smoked over time. Overall, participants described feeling “captivated” by the content and expressed eagerness to learn more.

### Step 6: Developing Full Functionality

Building upon the prototype as the foundation, we applied insights from the earlier steps to develop a full program consisting of 42 days of 2- to 3-minute focused conversations. These conversations were distributed over several phases of treatment: a prequit phase (14 days of content), quit day (1 day of content), and postquit phase (27 days of content). There are also conversations for those not ready to quit smoking by day 14 (6 days of content) and conversations for those who have relapsed (3 days of content). The content, described in Textboxes 1 and 2, follows US Clinical Practice Guidelines for cessation interventions [62]. The program content was presented as a continuous conversation, built on user input from prior conversations. This ensures a personalized and adaptive approach based on the user’s stated motivations to quit, triggers to smoke, and number of cigarettes smoked. QuitBot was proactive and provided daily prompts to start a structured text conversation with Ellen at the user’s preferred time, such as “Hi Alex, are you free to chat?” Users also had the flexibility to reach out to Ellen at any time for on-demand help with urges, inspiration, mood, and slips.

**QuitBot and SmokefreeTXT (SFT): phases and corresponding content.**

**Phase (number of days) and content of both SFT and QuitBot**

- Prequit day (14 days)
  Triggers to smoke, motivations for quitting, setting a quit date, Food and Drug Administration–approved medication information, skills to be aware of and cope with urges, and cessation progress tracking
- Quit day (1 day)
  Encouragement and smoking status check-in
- Postquit (28 days)
  Withdrawal symptoms education, slips and relapse prevention, managing mood, managing cravings, and cessation progress tracking
- Not ready or quit date >14 days
  Reviews motivations for quitting and cessation progress tracking
- Anytime help
  Skills to cope with urges, mood, and slips

**Content communication.**

**How content is communicated:** **SmokefreeTXT (SFT) and QuitBot**

**Figure 3:**
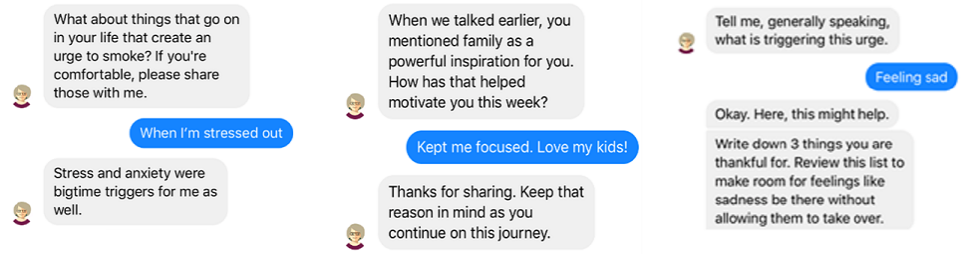
Representative functionalities of QuitBot include (A) determining triggers, (B) maintaining motivation, and (C) providing anytime urge help.

- SFT: sends texts of the content, answers to daily check-ins (eg, number of cigarettes smoked today), get 1-2 text responses, answers to entering anytime help keywords (eg, “CRAVE”), and get 1 text response
- QuitBot: digital coach sends the user a greeting to start a 2- to 3-minute conversation, presents content in a dialogue with the user via engagement features described in Figure 3 (eg, tailored responses and empathy), and answers to entering anytime help keywords (eg, “CRAVE”) initiate a dialogue

### Step 7: Programming QuitBot

We initially sought a development architecture with the flexibility to interact with QuitBot on any major consumer communication channel (eg, as a stand-alone app, FM, and Slack). Such flexibility adapts to current consumer trends in communication technology use, making QuitBot available for use on the channels with current high population-level reach. To determine which communication channel would be used for interacting with QuitBot, we conducted a web-based survey of 100 US adults who smoke, asking them which of these channels they would prefer for a chatbot: stand-alone app, WhatsApp (Meta Platforms, Inc), FM (Meta Platforms, Inc), Skype (Skype, Inc), or Slack (Slack Technologies, LLC). The majority of respondents (74/100, 74%) preferred FM, citing its familiarity, ubiquity, and ease of use. FM is an instant messaging service for online chats. At the time of the study (ie, 2019), there were >133 million FM users in the United States (1.3 billion globally) and FM hosts >300,000 chatbots, with 27% of them for health care (eg, exercise) [63-65]. Following these findings, we hosted QuitBot on FM.

Therefore, we custom built an architecture using the Microsoft Bot Framework that uses Microsoft Azure for the cloud computing and Microsoft Language Understanding (LUIS) platform for the natural language understanding of the QuitBot guide *Ellen*. The preference for natural language understanding over an if-then decision-based conversation flow was made to ensure a more natural and open-ended interaction, allowing a broad range of responses and better conveying that the user is being heard and understood. QuitBot’s LUIS allows it to understand common text shorthand. Users can respond freely or simply select from a menu of responses. If QuitBot does not understand a free response, it will say so and ask the participant to rephrase the response. QuitBot was written in the programming language of Node.js [66].

### Step 8: Conducting a Diary Study

We conducted a diary study to obtain ongoing feedback on users’ interactions with QuitBot, its design, and content. In user-centered design research, a diary study of 2 weeks with 6 to 12 participants is recommended to obtain this initial feedback [67,68]. Accordingly, we conducted a single-arm 14-day diary study of the program with 9 adults who were smoking at least daily (all smoked ≥30 cigarettes/d), were interested in quitting smoking, and recruited from around the United States via Facebook advertisements. Four were chosen because they were skeptical about chatbots being able to help someone quit smoking, while the remaining 5 were neutral about them. Participant demographics were as follows: mean age 40.4 (SD 13.4) years, 11% (1/9) from racial and ethnic minority backgrounds, 44% (4/9) female, and 67% (6/9) had less than a bachelor’s degree.

All 9 participants completed the following: (1) the 60-minute video-based orientation focusing on how to use QuitBot and complete the daily diary entries; (2) 14 evening diary entries (15 min each) about their daily interactions with QuitBot, its design, and content; (3) on day 7, a midpoint 15-minute video call with a member of our user research team to review their impressions to date; and (4) a 60-minute video call exit interview with a member of our user research team. A PhD-level UX researcher with >20 years of experience conducted the interviews. (Example questions from the exit interview are as follows: “Which parts of the app did you find the most helpful? Why?”) Semistructured interviews were conducted in person at the lead author’s UX Research HABIT laboratory. The deductive thematic analysis organized the user’s responses by grouping them into themes, reviewing the themes, and then interpreting them [56-58].

The results showed that, although the focus was on usability, by day 14, three participants quit smoking and the remaining 6 participants reduced to 3 or 4 cigarettes per day. Ratings for usefulness (“Overall, how useful was the QuitBot app for helping you quit smoking?”), satisfaction (“Overall, how satisfied were you with the QuitBot?”), and likelihood of recommending QuitBot (“To what extent would you recommend QuitBot to someone who would like to quit smoking?”) were all high: 4.33, 4.67, and 4.88, respectively, on a 0 (not all) to 5 (extremely) scale. All 9 users felt highly supported by Ellen and liked her persona. They liked the skills training for coping with smoking urges and lapses. Their feedback yielded minor content edits and fixes of technical bugs. Representative functionalities of QuitBot are shown in Figure 3.

### Diversity and Inclusion in UX Design

The diversity of race, gender, age, and educational characteristics of users who participated in our UX design studies influenced the design in many ways, including Ellen’s persona design (eg, men and women both preferred a female persona), Ellen’s stories of people who have quit smoking (eg, they were diverse in age, gender, race, and education), use of language (eg, fifth-grade reading level, informal, and respectful), and user interface (eg, larger response buttons and larger font size [69]).

### Step 9: Conducting a 3-Arm Pilot RCT

The favorable feedback from the diary study led us to conduct a 3-arm parallel pilot RCT comparing QuitBot (n=200) to the SmokefreeTXT (SFT; n=149) intervention and to a QuitBot delayed access control group (n=55). Following expert recommendations for pilot RCT design [70,71], the feasibility outcomes were the study’s primary focus to inform the further development of QuitBot and design of a future full-scale trial of QuitBot. As this pilot RCT was the first time QuitBot was tested and no prior RCTs had been reported on any quit smoking chatbot, estimated effect sizes were unknown. Instead, the sample sizes were based on comparable sample sizes from prior pilot studies we had conducted in our laboratory [72,73]. Participants were recruited nationwide and were randomized to the intervention arm using randomly permuted blocks of size 2, 4, and 6, stratified by biological sex (male vs female), heaviness of smoking index score (≤4 vs >4), and percent confidence in being smoke-free in 12 months (≤70% vs >70%). The study was double-blinded, with both interventions called “QuitBot.”

### Ethical Considerations

All study procedures were approved by the Fred Hutch Cancer Center Institutional Review Board (8659/RG1001766). The clinical trial protocol was approved by the Fred Hutch Scientific Review Committee (FHIRB008659), and the trial was registered on ClinicalTrials.gov (NCT03585231). There were no deviations to the registered protocol. All study participants provided informed consent, and data were deidentified for privacy and confidentiality.

### Eligibility Criteria for the Pilot RCT

The inclusion criteria were as follows: (1) age ≥18 years; (2) having smoked at least 1 cigarette a day for at least the past 12 months; (3) wanting to quit cigarette smoking within the next 14 days; (4) if concurrently using any other nicotine or tobacco products, wanting to quit using them within the next 14 days; (5) being interested in learning skills to quit smoking; (6) being willing to be randomly assigned to either condition; (7) residing in the United States; (8) having daily access to their own smartphone; (9) having both SMS text messaging and FM on their smartphone (criteria 8 and 9 were required to receive each interventions’ content); (10) being willing and able to read in English; and (11) not using other smoking cessation interventions. Individuals deemed ineligible to participate were directed to the smokefree.gov website and the 800-QUIT-NOW number for access to their state’s quitline resources.

### SFT Comparison Condition

**Figure 4:**
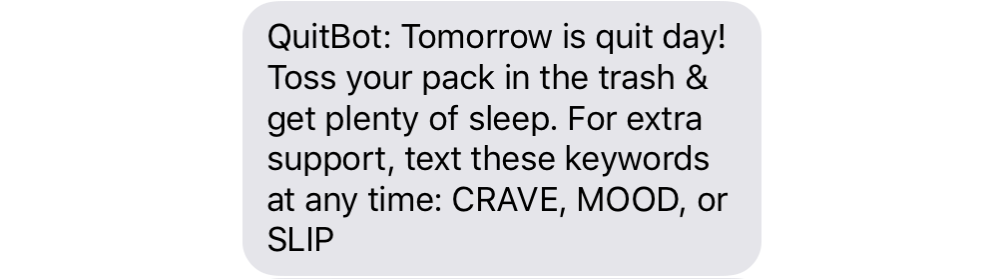
Sample SmokefreeTXT text message.

For the past 20 years, mobile phone–delivered SMS text messaging interventions have been a prominent technology for delivering smoking cessation interventions [74-78]. Each year, SMS text messaging smoking cessation interventions are reaching >300,000 US adults who smoke and 6 million adults who smoke worldwide [79,80]. SFT’s 42-day program was developed by the National Cancer Institute (NCI). SFT is the most widely accessible SMS text messaging program in the United States. SFT is nonproprietary and free to the public, thereby providing maximal accessibility and replicability. Daily messages are sent about the importance of quitting smoking, setting a quit date, preparing to quit, quitting, and maintaining abstinence. Daily messages check in about quit status. Three keywords can be proactively sent by users to receive help anytime: “CRAVE” (on how to cope with urges), “MOOD” (on how to cope with moods triggering smoking), and “SLIP” (on how to cope with lapses). Participants do not need to respond to or otherwise engage with SFT messages to complete the SFT program. Refer to Figure 4 for sample messages.

NCI’s SFT contractor (ICF International [81]) provided us with the full content of SFT so that we could internally host a secured private version for research. In both SFT and QuitBot, participants receive 2 prompts per day (3 on the quit day). Comparisons between QuitBot and SFT are shown in Textboxes 1 and 2.

### QuitBot Delayed Access Comparison Condition

To explore the unique impact of QuitBot on smoking cessation, considering that some participants might quit smoking without intervention, we introduced a delayed access comparison condition. In this condition, 55 participants received delayed access to QuitBot after completing the 3-month follow-up survey. The delayed access comparison condition served the ethical purpose of providing participants access to a treatment (as opposed to no treatment at all).

### Outcome Measures

Outcome data were collected through an encrypted web-based survey. Participants not completing the web-based survey were sequentially offered the survey via phone, mailed survey, and postcard. The primary feasibility outcomes were (1) sufficient accrual of the planned number of study participants, (2) balanced demographic and smoking characteristics at baseline between study arms, and (3) retention of the primary 30-day PPA smoking outcome at the 3-month follow-up. Intervention engagement was assessed based on comparing the active treatment study arms on the number of times and number of days participants interacted with their assigned intervention. All interactions with the participants’ assigned interventions were objectively logged using an internally hosted secure server. The primary smoking cessation outcome was 30-day PPA, based on compete-case analysis, and 7-day complete-case PPA was secondary.

### Statistical Analysis for the Pilot RCT

The feasibility of the pilot RCT was assessed based on sufficient accrual, balanced randomization, and adequate follow-up data retention rates that did not differ between arms. Baseline characteristics were compared between the 3 study arms using ANOVA for continuous variables and Fisher exact tests for categorical variables and were summarized with the “arsenal” package in R (version 4.2.3; R Foundation for Statistical Computing) [82,83]. We used generalized linear models to assess differences between study arms in the number of days participants used their intervention.

We used negative binomial models, implemented with the R package “MASS” [84], to compare treatment arms on total number interactions because the data were heavily right-skewed. Logistic regression models were used to test the effect of the treatment arm on binary smoking cessation outcomes. On the basis of evidence from meta-analyses of SMS text messaging trials [85], all outcome models were adjusted for the 3 factors used in stratified randomization: biological sex (male vs female), heaviness of smoking index score (≤4 vs >4), and percent confidence in being smoke-free in 12 months (≤70% vs >70%). Wald tests for pairwise comparisons of each outcome between study arms were adjusted for multiple comparisons with the Holm procedure [86]. Statistical tests were considered significant at α<.05. Deductive thematic analysis organized participants’ comments about QuitBot by grouping them into themes, reviewing the themes, and then interpreting them [56-58].

## Results

### Step 10: Main Results of the Pilot RCT

#### Recruitment Was Successful

On the basis of our successful methods for national recruitment [87], we developed and tailored Facebook advertisements with ongoing monitoring and adjustment of recruitment yield. These efforts resulted in screening 2954 participants, with 1380 eligible, 583 consenting, and 418 randomized between September 2018 and June 2019. After the completion of study participation, 14 participants were found to be cases of fraud, duplicate participants, or in the same household as another participant, leading to a total of 404 participants included in analyses.

#### Randomization

The 3 stratification conditions were balanced at baseline on all measured characteristics (all *P* values >.05). As shown in Table 1, participants were on average 36 years old, 70% (283/404) were women, 28.9% (116/401) reported being from racial or ethnic minority backgrounds, 52.7% (213/404) were unemployed, 83.9% (339/404) had no college degree, 71.5% (289/404) smoked more than one-half pack daily, and 59.9% (242/404) had high cigarette dependence (Fagerström Test for Cigarette Dependence scores of ≥6). The characteristics of this FM sample are very similar to those of other digital health intervention trials [85,88,90].

**Table 1:** Baseline participant characteristics by study arm.

Characteristic					Total (n=404)		SmokefreeTXT (n=149)		Delayed (n=55)		QuitBot (n=200)		*P* value	
**Baseline characteristic**														
	Age (y), mean (SD)				36.0 (10.4)		36.2 (11.2)		35.6 (9.6)		35.9 (9.9)		.92	
	**Gender, n (%)**													
		Woman		283 (70)		103 (69.1)		39 (70.9)		141 (70.5)		.95		
		Man		121 (30)		46 (30.1)		16 (29.1)		59 (29.5)				
	**Race, n (%)**													.52
		Asian		2 (0.5)		0 (0)		0 (0.0)		2 (1)				
		Black or African American		51 (12.6)		21 (14.1)		9 (16.4)		21 (10.5)				
		Native American or Alaska Native		12 (3)		4 (2.7)		0 (0)		8 (4)				
		Native Hawaiian or Pacific Islander		1 (0.2)		0 (0)		0 (0)		1 (0.5)				
		White		296 (73.3)		110 (73.8)		40 (72.7)		146 (73)				
		Multiple races		31 (7.7)		13 (8.7)		4 (7.3)		14 (7)				
		Unknown race		11 (2.7)		1 (0.7)		2 (3.6)		8 (4)				
	Hispanic ethnicity, n (%)				28 (6.9)		7 (4.7)		6 (10.9)		15 (7.5)		.27	
	Minority race or ethnicity (n=401), n (%)				116 (28.9)		42 (28.2)		17 (30.9)		57 (28.9)		.93	
	Married, n (%)				104 (25.7)		32 (21.5)		16 (29.1)		56 (28)		.32	
	Employed, n (%)				191 (47.3)		80 (53.7)		24 (43.6)		87 (43.5)		.14	
	No college degree, n (%)				339 (83.9)		126 (84.6)		47 (85.5)		166 (83)		.87	
	Heavy alcohol use (n=395), n (%)				47 (11.9)		18 (12.5)		6 (11.3)		23 (11.6)		.96	
	Positive depression screening results (n=402), n (%)				223 (55.5)		91 (61.5)		28 (50.9)		104 (52.3)		.17	
**Smoking behavior**														
	FTCD^a^ score, mean (SD)				5.7 (2.0)		5.5 (2.0)		6.1 (2.2)		5.7 (2.0)		.17	
	High nicotine dependence, n (%)				242 (59.9)		88 (59.1)		36 (65.5)		118 (59)		.66	
	Smokes more than one-half pack per day, n (%)				289 (71.5)		98 (65.8)		42 (76.4)		149 (74.5)		.14	
	Smokes >1 pack per day, n (%)				66 (16.3)		20 (13.4)		14 (25.5)		32 (16)		.11	
	First cigarette within 5 minutes of waking, n (%)				205 (50.7)		75 (50.3)		34 (61.8)		96 (48)		.19	
	Smoked for ≥10 years, n (%)				317 (78.5)		112 (75.2)		44 (80)		161 (80.5)		.46	
	Used e-cigarettes at least once in the past month, n (%)				122 (30.2)		42 (28.2)		16 (29.1)		64 (32)		.73	
	Quit attempts in the past 12 months (n=377), mean (SD)				1.6 (4.7)		1.6 (3.3)		1.1 (3.2)		1.7 (5.8)		.68	
	At least 1 quit attempt in the past 12 months (n=377), n (%)				145 (38.5)		51 (37.8)		16 (30.2)		78 (41.3)		.33	
	Confidence to quit smoking, mean (SD)				64.1 (27.0)		62.6 (27.0)		72.2 (27.3)		62.9 (26.8)		.05	
	**Friend and partner smoking**													
			Close friends who smoke, mean (SD)	2.8 (1.7)		2.8 (1.7)		2.7 (1.6)		2.8 (1.8)		.97		
			Number of adults in home who smoke, mean (SD)	1.5 (0.9)		1.4 (0.9)		1.7 (1.1)		1.5 (0.8)		.19		
			Living with partner who smokes, n (%)	145 (35.9)		51 (34.2)		24 (43.6)		70 (35)		.43		

^a^FTCD: Fagerström Test for Cigarette Dependence.

#### The 3-Month Follow-Up Rates Were High

To maximize outcome data completion, we followed our team’s successful protocol [87]: 4 sequential survey modalities (first web, followed by phone, mail, and postcard). As agreed in the informed consent, participants received US $25 for submitting their responses and received an additional US $10 bonus for completing the web survey within 24 hours. The achieved *outcome survey completion rate of 96%* provided confidence in the follow-up survey methods. The data retention did not differ between study arms (*P*=.54). Given the limitations of the pilot budget, cessation data were self-reported.

#### Engagement and Cessation Results Were Promising for QuitBot

The number of times participants interacted with their assigned intervention was 1.3 times greater in QuitBot as compared to SFT (incidence rate ratio 1.33, 95% CI 1.04-1.70; *P*=.02; Table 2). Participants used their assigned intervention 11 days longer in the QuitBot arm than in the SFT arm (point estimate 11.5, 95% CI 4.9-18.1; *P*=.001). QuitBot’s intervention completion results are substantial when considering that each day’s content involved a 2- to 3-minute conversation. (By contrast, SFT participants did not need to respond to or otherwise engage at all with their messages to complete their program; daily SFT text messages were sent automatically.) Participant engagement was limited by QuitBot’s inability to answer participants’ open-ended questions (see the *Representative QuitBot Comments* section). Therefore, cessation results are reported for all participants and for participants who completed their assigned intervention.

For all participants, the 30-day PPA rates at 3-month follow-up were 31.1% (59/190) for QuitBot versus 34.7% (50/144) for SFT (QuitBot vs SFT: odds ratio [OR] 0.81, 95% CI 0.50-1.29; *P*=.36; Table 3) versus 7% (4/54) for delayed treatment (QuitBot vs delayed: OR 5.97, 95% CI 2.04-17.45; *P*=.002). For those who completed their assigned intervention (ie, viewed all 42 days of planned content), the 30-day, complete-case, PPA rates at 3-month follow-up were 63% (39/62) for QuitBot versus 38.5% (45/117) for SFT (QuitBot vs SFT: OR 2.58, 95% CI 1.34-4.99; *P*=.005). The pattern of results was highly similar for the outcome of 7-day, complete-case, PPA rates at 3-month follow-up, albeit with higher abstinence rates in each study arm.

**Table 2:** Comparison of QuitBot and SmokefreeTXT (SFT) interventions on 3-month engagement outcomes.

Study engagement outcome	SFT (n=149), mean (SD; median)	QuitBot (n=200), mean (SD; median)	QuitBot vs SFT			
			IRR^a^ (95% CI)	*P* value	PE^b^ (95% CI)	*P* value
Number of times interacted (n=266)	24.2 (25.8; 15)	32.9 (29.0; 25)	1.33 (1.04-1.70)	.02	—^c^	—
Days from randomization to last input	44.1 (22.7; 54)	55.7 (36.0; 70)	—	—	11.5 (4.9-18.1)	<.001

^a^IRR: incidence rate ratio.

^b^PE: point estimate.

^c^Not applicable.

**Table 3:** Comparison of QuitBot and SmokefreeTXT (SFT) interventions and delayed intervention on 3-month cessation outcomes.

Study outcome	SFT (n=149), n (%)	Delayed^a^ (n=55), n (%)	QuitBot (n=200), n (%)	QuitBot vs SFT		QuitBot vs delayed^a^	
				OR^b^ (95% CI)	*P* value	OR (95% CI)	*P* value
30-day cigarette abstinence among all participants (n=388)	50 (35)	4 (7)	59 (31)	0.81 (0.50-1.29)	.36	5.97 (2.04-17.45)	.002
30-day cigarette abstinence among program completers (n=179)	45 (38)	—^c^	39 (63)	2.58 (1.34-4.99)	.005	—	—
7-day cigarette abstinence among all participants (n=388)	76 (53)	5 (9)	91 (48)	0.79 (0.51-1.22)	.28	10.08 (3.79-26.80)	<.001
7-day cigarette abstinence among program completers (n=179)	70 (60)	—	50 (81)	2.63 (1.24-5.55)	.01	—	—

^a^Three-month delay in receiving QuitBot.

^b^OR: odds ratio.

^c^Not applicable.

#### Representative QuitBot Comments

Comments from QuitBot arm trial participants reflected a strong overall bond with the chatbot’s persona:

I loved Ellen. She was always there when I needed her.

Ellen was always there for me when I had a craving.

I love how engaged she was, I could really quit with her there to talk to.

She made me feel like I was not alone.

She was there without making me feel ashamed.

She was kind, nonjudgmental.

She held me accountable.

Felt like a friend encouraging me.

Conversely, participants were frustrated by QuitBot’s inability to respond to their specific questions about quitting smoking:

I could not ask questions and get real answers back.

I could not ask it real live questions.

I wanted to write my own questions.

Can’t ask any question.

Not being able to respond to my questions.

I wish you could talk to her...without it being a constant couple of options.

I didn’t like how it selected responses.

The fact that you cannot ask a question and [it] has no idea what you are saying unless you select one of the options.

#### Main Conclusions From the Pilot RCT

Our main conclusions were as follows: (1) the intervention demonstrated potential for rigorous testing based on sufficient accrual, balanced randomization, and high retention rates; (2) overall, there was a strong engagement with QuitBot; and (3) promising quit rates were observed, particularly among participants who completed the content of their assigned intervention. The effectiveness of QuitBot was evident, as quit rates in the delayed condition group were significantly lower (59/190, 31.1% vs 4/54, 7%; *P*=.002), indicating a net percentage point increase in smoking cessation of 24%. Therefore, it is highly unlikely that effects of QuitBot were merely due to the passage of time or baseline motivation to quit smoking (ie, few participants quit without offering intervention).

Challenges were also identified, potentially impacting participant engagement and quit rates. Specifically, QuitBot’s inability to respond to participants’ own questions about quitting smoking led to a significant level of frustration. While the participant can answer questions asked by the QuitBot (eg, “Tell me what is triggering your urge.”), the reverse was not possible: participants could not ask QuitBot their own questions. A QuitBot feature that allows participants to ask free-form questions would be needed to address this limitation.

#### Technical Limitations of the FM Platform

While FM was the preferred communication platform from our survey results, Facebook introduced changes that would limit participants’ engagement with QuitBot as well as our own access to user data: (1) Facebook made policy changes that revoked access permissions to proactively outreach (eg, to invite participant to check in or start a conversation), effectively removing our ability to proactively contact users (restricting that ability to news-related apps only); and (2) Facebook made platform changes that restricted our ability to access demographic information of users, inhibiting data collection. Facebook’s changes raised concerns about the feasibility of QuitBot’s conversational functionality and data collection. This critical limitation could be addressed by transitioning to a stand-alone smartphone app communication platform, enhancing accessibility and control for both participants and the development and research teams.

### Step 11: Building a Main Function Enabling Users to Pose Free-Form Questions About Smoking

#### Overview

The goal of this specific QuitBot refinement was to build a main function of QuitBot that would enable users to pose free-form questions about quitting cigarette smoking and for the QuitBot to respond with accurate, concise, professional, and nonrepetitive answers. This was an iterative 3-step process, which is detailed in Table 4.

**Table 4:** Steps, sources, and results of QuitBot’s question and answer (QnA) iterative development process.

Step	Source (year)	Results
1. Generate QnA pairs	Alexander Street therapy transcripts (2020) National Cancer Institute call center transcripts (2020) HABIT laboratory cessation counseling intervention transcripts (2020) HABIT laboratory digital intervention content (2020) HABIT laboratory clinical team generates QnA (2020-2021) Prolific survey of adults who Smoke (2021)	11,000 smoking QnA pairs 8223 chitchat QnA pairs
2. Training LLM^a^ models on QnA pairs	Azure application programming interface (2020-2023) DialoGPT (2021) ParlAI (2021) Davinci GPT-3 (2021) Curie GPT-3 (2021) Ada GPT-3 (2021) Contextualized GPT-3.5 (2022) GPT-4.0 (2023)	Models with higher self-scored confidence about answers provided: processed by Azure application programming interface Models with lower self-scored confidence about answers provided: handled by Curie GPT-3 (2021) and replaced by contextualized GPT-3.5 (2022)
3. Evaluating LLM outputs	Automated evaluation: pertinence and grammaticalness (2020-2023) Manual evaluation: accuracy and tone (2021-2023) 14-day user resting (2021)	Identified the answers that were repetitive, incorrect, or had impersonal or nonprofessional tone

^a^LLM: large language model.

#### Step 11.1. Generating QnA Pairs

The first step was to develop a knowledge base of QnA pairs focused on the topics of quitting cigarette smoking. Smoking cessation clinical data sources included the Alexander Street data sets of counseling transcripts [90], NCI call center transcripts of smoking cessation coaching conversations [91], and transcripts of counseling sessions from our Fred Hutch Cancer Center research laboratory’s prior smoking cessation intervention trials [53]. The categories of smoking cessation questions spanned a wide range, including medications to aid smoking cessation, the role of vaping e-cigarettes in quitting smoking, health consequences of smoking on self and others, staying motivated to quit smoking, triggers to smoke, barriers to quitting smoking, tips for managing cravings and withdrawal, and relapse prevention and recovery.

For generating a diversity of QnA sources, the knowledge base was broad, drawn from Alexander Street transcripts of therapy sessions [90], NCI call center transcripts of web-based Live Chats [91], Fred Hutch Cancer Center’s HABIT laboratory cessation counseling intervention transcripts [53], written clinical content from HABIT laboratory digital smoking cessation interventions [92,93], and manual generation of questions and answers by HABIT laboratory clinical team members. We created a sequence-to-sequence (seq2seq) training model and processed cleaned transcripts, generating >8000 QnA pairs specifically focused on the topic of quitting cigarette smoking. As detailed in step 3, our evaluations led us to revisit step 1. In this iteration, we generated 2000 new questions posed by 32 adults interested in quitting cigarette smoking in a Prolific web-based survey. Then, our HABIT laboratory clinical team members generated answers to those questions. We also generated 1000 additional new questions from NCI call transcripts and transcripts related to our laboratory’s intervention research. This iterative process yielded >11,000 QnA pairs.

#### Step 11.2. Training LLMs on QnA Pairs

The second step was to use these QnA pairs to train a series of LLMs. The first LLM deployed was an Azure application programming interface customized for the chatbot’s 6-level chat architecture:

1. Motivations to quit smoking, triggers to smoke, and frequency of smoking
2. Dispatcher dialogue flow, to interpret and route utterances to the correct target, either Azure’s 8000 QnA library or a custom AI model
3. QnA-maker-based “caring” personality chitchat to express empathy and provide supportive interactions with the user
4. QnA-based custom “help” to respond to trigger words such as suicide
5. Custom transformer-based model to handle chitchat QnA scenarios in dialogues that do not align with any specific intent
6. Custom transformer-based model designed to address smoking-related QnA scenarios in dialogues that do not align with any specific intent

In 2021, we tested 4 neural network models: a preexisting DialoGPT “Chitchat” model, 2 smoking content–trained DialoGPT models (1 trained on our 8000 QnA pairs and 1 on NCI chatlogs), and 1 smoking content–trained ParlAI model. We then accessed OpenAI’s GPT-3 and conducted a comparative analysis of its model performance against our then best-performing model DialoGPT. GPT-3 outperformed the specially trained DialoGPT. Subsequently, to further improve GPT-3 performance, we compared the following model options: (1) Davinci, (2) Curie, and (3) fine-tuned Curie, concluding that the fine-tuned Curie model provided the best performance. Upon the release of GPT-3.5 (March 2022), we compared its performance with the fine-tuned GPT-3 Curie model [94]. This evaluation involved batch testing of questions that were not present in the QnA library, followed by a ranked review of answers (Textbox 3). GPT-3.5 provided better or comparable answer quality but required additional contextualizing parameters to match fine-tuned performance tailored for smoking-specific content.

Therefore, we used GPT-3.5 to expand the Azure QnA library by generating variations of existing questions and answers. This procedure expanded the Azure QnA library’s capacity to accommodate a wider range of user question formulations, providing QuitBot with greater control over the quality of answers delivered to users.

**Comparisons of large language model answers to 3 sample questions on quitting smoking.**

**Sample question 1: “What is the quickest way to stop smoking?”**

- Davinci: “The quickest way to stop smoking is to not start!”
- Curie: “Tapering down is the best way to stop smoking. Quitting smoking is a process. Make a quit plan and notice what triggers you to smoke.”
- Fine-tuned Curie: “Our program was created to give you a plan that will work for you. We start by making a quit plan. Write down what inspires you to quit smoking. Choose a quit date and start cutting back on cigarettes.”
- GPT-3.5: “The quickest way to stop smoking is to quit cold turkey, which means stopping smoking abruptly and without any aids such as nicotine replacement therapy or medication. However, quitting cold turkey can be difficult and the withdrawal symptoms can be challenging. We found that it works best to follow the program steps: make a quit plan and notice what triggers you to smoke. Then use quit smoking medication to help with withdrawal and use our tools to help urges pass as you quit gradually. Either way, it’s important to find a method that works for you and stick with it.”

**Sample question 2: “I smoke when I am lonely. What should I do?”**

- Davinci: “You are addicted to the nicotine in cigarettes. The only way to truly stop smoking is through nicotine replacements or prescription medications.”
- Curie: “It’s hard to feel lonely. Notice what thoughts and feelings are there and try not smoking them away.”
- Fine-tuned Curie: “The first step is to make a quit plan. Write down what inspires you to quit smoking. Choose a quit date and start cutting back on cigarettes. Be aware of what triggers you to smoke.”
- GPT-3.5: “It’s hard to feel lonely. Notice what thoughts and feelings are there and try not smoking them away. Trying to be a friend to yourself in that moment and do something that would be nourishing to you.”

**Sample question 3: “Aren’t e-cigarettes like the nicotine inhaler?”**

- Davinci: “No, because unlike nicotine inhalers, e-cigarettes are used as simultaneous consumption of nicotine and air.”
- Curie: “No. While e-cigarettes/vaping don’t have the harmful contaminants of tobacco smoke they are not safe.”
- Fine-tuned Curie: “They appear similar but are actually very different. The content of the nicotine inhaler is regulated by the FDA and when used stays mostly in throat and mouth. E-cigarettes are less harmful than regular cigarettes however, they are still addictive and contain noxious chemicals which can damage the heart and blood vessels.”
- GPT-3.5: “Although they may look similar, the content of nicotine inhalers and e-cigarettes are actually different. The nicotine inhalers are regulated by the FDA and the nicotine content stays mostly in the throat and mouth. The e-cigarettes are not regulated, and the nicotine content can vary greatly, sometimes having more nicotine than a cigarette. They can also have other harmful substances besides nicotine. Because it is heated into a mist, the vapor goes into the lungs.”

#### Step 11.3. Evaluating LLM Outputs

The third step in the development of the QuitBot’s QnA feature involved evaluating the results of the LLMs using both automated and manual evaluation methods as well as user testing from adults who wanted to quit smoking. The automated evaluation used a scoring system of entropy (pertinence) and perplexity (grammaticalness) scores to rate the quality of answers generated by the LLMs. In addition, we implemented a filtering and scoring system to enhance the QuitBot’s ability to communicate when it fails to comprehend a question. In such cases, the QuitBot asks users to be more specific if the confidence score for an answer falls below a certain threshold. The manual evaluation was conducted by trained raters in our HABIT laboratory, who hand scored the results of the models on measures of answer *accuracy* (yes or no), *repetitiveness* (yes or no), and *tone* (acceptable or needs improvement). Answers requiring improvement underwent manual revisions and were included into future iterations of model testing.

After retraining the model, in 2022, adults who smoke (n=14) were recruited from nationally placed Facebook advertisements to provide feedback on QuitBot prototype over a period of 14 days. Participant demographics were as follows: mean age 44.6 (SD 9.9) years, 43% (6/14) from minority race and ethnicity backgrounds, 64% (9/14) female, and 50% (7/14) had less than a bachelor’s degree. A PhD-level UX researcher with 20 years of experience conducted the interviews. Deductive thematic analysis organized the user’s responses by grouping them into themes, reviewing the themes, and then interpreting them [56-58].

**Figure 5:**
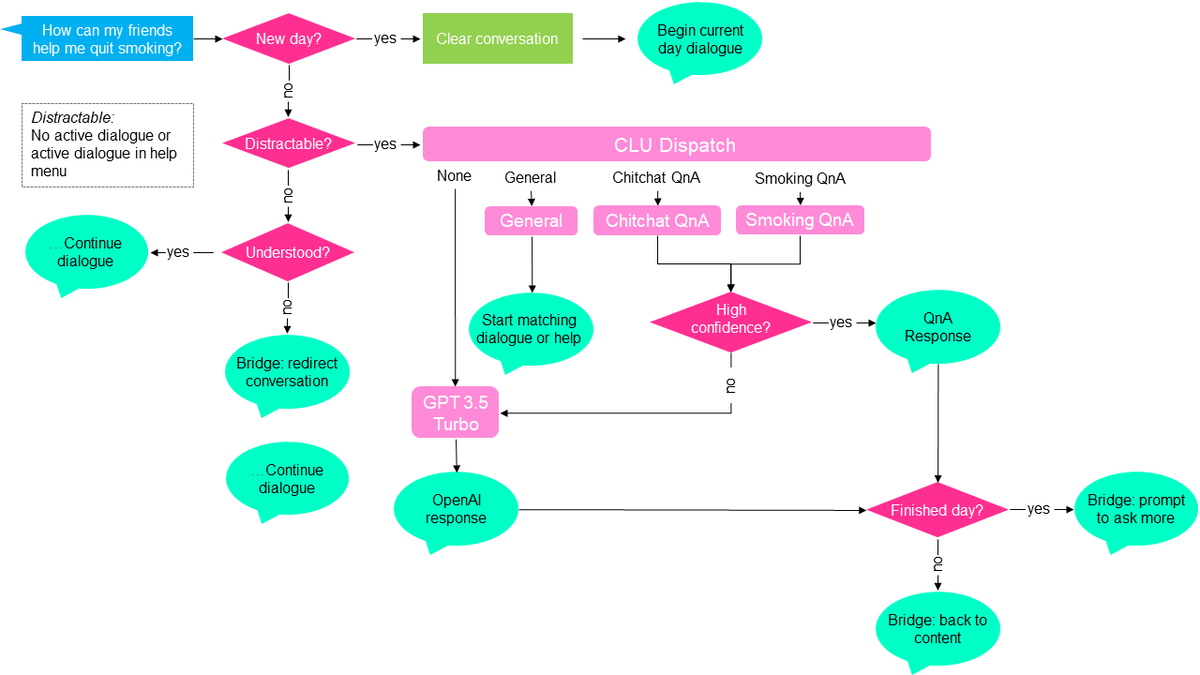
QuitBot’s architecture for handling free-form questions about quitting smoking. CLU: conversational language understanding; QnA: question and answer.

Participants were highly receptive to the structured clinical conversations and noted that the free-form chats required additional fine-tuning to enhance comprehension of the users’ questions. On the basis of this feedback, we determined the final organization of the chatbot architecture, combining 2 different models (Figure 5):

1. Microsoft’s Azure QnA maker to handle questions matching our library of 11,000 QnA pairs that are based on scientific and clinical expertise. QnA maker uses Microsoft Language Understanding framework (LUIS) to leverage transformer models for responding to structured questions, with vetted answers stated in a professional manner.
2. Fine-tuned GPT-3.5 Turbo model to respond to questions that are not within our library of QnA pairs. Answers accurately with human-like variability, with different wording variations each time. GPT-3.5 is a backup to our QnA library.

From the perspective of computer science, the QuitBot’s chatbot’s program runs on a *finite state machine* [95], which is a model of a system that runs on a limited set of modes. Depending on which mode the program is in, the QuitBot will behave in one manner or another. Ellen’s state machine tracks what it is currently doing and combines interactions with the user to determine the next state. As the user can say anything, a hierarchy of possible intentions ranked by importance to the current state is used to decide the response to the user and the next state of the conversation.

A daily welcome exchange is an example. In this example, it is the start of the day and Ellen is in the “welcome” state. The user opens the app and says, “Hi!” To handle this prompt, the user’s text goes through several steps in the finite state machine, illustrated in Figure 5: (1) categorize the user’s intent; (2) determine if the intent is relevant to the current state of the conversation; (3) accordingly, move the current state of the conversation; and (4) formulate a response. In this case, “Hi!” is interpreted as a greeting intent, which is relevant to the current conversation. Ellen moves the state of conversation to “daily check-in” and responds with a greeting of her own, “Good morning. Thanks for checking in.” When formulating a response, the user’s intent determines which AI model will be used. General banter goes to a “chitchat” model powered by our Azure QnA library, smoking questions go to the Azure QnA model specifically trained on smoking questions, and unknown or low confidence intents go to GPT-3.5 Turbo. Finally, prewritten responses from our Azure QnA library that fit into the current conversation are used for specific scenarios.

### Final Version of QuitBot

The final version of QuitBot is a stand-alone app that features (1) a personal coach (named “Ellen”) who supports the user; (2) a series of 42 days of 2- to 3-minute structured clinical conversations with Ellen, guiding the user through distinct stages of quitting smoking; and (3) the ability for users to pose any free-form question related to quitting smoking. The structured conversations provide the valuable function of a clear step-by-step program for staying motivated, learning about one’s triggers to smoke, setting a quit date, and staying smoke-free. Complementing the structured conversations, the free-form question feature provides users the freedom to ask their own questions, the option to address unique clinical needs, and the opportunity to follow-up on the content provided in the structured conversations. The combination of both structured and free-form conversation features is intended to balance their main strengths and limitations: the structured clinical format offers a guided program on quitting smoking, albeit with limited user question flexibility, while the open-ended format provides freedom but may encounter instances of not fully understanding the user’s questions to give them clear guidance, despite the positive performance of the QnA feature thus far. Representative screenshots of QuitBot are provided in Multimedia Appendix 1.

## Discussion

### Principal Findings

This paper described the research group’s 4-year process of developing a conservational chatbot for cigarette smoking cessation (“QuitBot”). The user-centered development process yielded a comprehensive quit smoking program that follows a series of 42 days of 2- to 3-minute structured clinical conversations. The program content covers topics ranging from motivations to quit, setting a quit date, choosing FDA-approved medications, identifying and coping with a wide range of triggers to smoke, and recovering from lapses or relapses. The program content, which follows the US Clinical Practice Guidelines for smoking cessation, is presented as a continuous conversation, built on user input from prior conversations. QuitBot is available for both proactive and on-demand assistance at any time. Users can continue to interact with QuitBot after completing the 42 days of conversations.

Pilot RCT testing of QuitBot showed that the intervention had high user engagement and promising cessation rates, especially among participants who completed their assigned intervention. However, Facebook made policy changes that revoked access permissions to proactively outreach (eg, to invite participant to check in or start a conversation), effectively removing our ability to proactively contact users (restricting that ability to news-related apps only). This limitation was addressed by changing the FM communication platform to a stand-alone smartphone app that is fully accessible and controllable by the development and research teams.

The primary feedback from users was their frustration that the QuitBot could not respond to their own questions about quitting smoking. Therefore, we created the core conversational feature that would allow users to ask free-form and open-ended questions about quitting cigarette smoking and for the QuitBot to respond with accurate, concise, professional, and nonrepetitive answers. We developed a library of 11,000 QnA pairs on the topic of quitting cigarette smoking. The results of our model testing showed that Microsoft’s Azure-based QnA maker could handle any question that matched our library of 11,000 QnA pairs. In contrast, a fine-tuned, contextualized GPT-3.5 could answer new questions that were not within our library of QnA pairs.

QuitBot has several key limitations that might present a challenge for users who expect fast responses to their questions. QuitBot was designed for users to wait until the end of the 2- to 3-minute structured clinical conversations before they can ask free-form questions. This design element was necessary to prevent breaking the logic of each of the structured conversations and thereby going off on tangents without an ability to return to the structured conversation. We address this design element by asking the user to hold onto their questions until the end of the structured conversation at various times throughout the program. To date, this message appears to have been effective at training the user to wait until the end of the structured conversation to ask free-form questions. The second major limitation is the response time latency for free-form questions when the GPT servers are running at capacity. While usually the response latency is only a few seconds, we have observed some instances where it can take up to 30 seconds. To address this potential delay, we inform users that it may take a moment to answer their questions and appreciate their patience.

By contrast, this study has numerous strengths that have the potential to advance clinical intervention development research and practice to aid smoking cessation. Most importantly, this study illustrates the value of following a methodical, user-centered design framework in the development of technology interventions. The framework has yielded a chatbot with a comprehensive step-by-step clinical program for quitting smoking and possesses a broad knowledge base on the topic of quitting smoking. QuitBot allows users to ask free-form and open-ended questions about quitting smoking, with answers informed by a broad set of clinical experience and scientific research. This technical capability has been afforded by the LLMs that underlie the state-of-the-art versions of Azure QnA Maker and GPT. The result is that users can obtain accurate and informative answers to their questions, which would otherwise be difficult to glean and evaluate from other accessible digital resources such as internet searches.

By contrast, prior reports of chatbots only address certain aspects of the quit smoking process, such as providing reflections on the pros and cons of smoking or helping ambivalent adults who smoke in contemplating a quit attempt. Only 8% of participants rated such chatbots as helpful [36]. Similarly, early iterations of QuitBot, which relied on a forced-choice answer format, left participants wishing responses tailored to their individual needs [96]. Likewise, a study of 6 users of a tablet-based chatbot, aimed at encouraging them to contemplate quitting and set a quit date, was limited by a forced-choice answer format [97]. In the only prior RCT of a smoking cessation chatbot, responses were confined to preset scripts and had an outcome data retention rate of only 45% [98].

Although users are informed that QuitBot is only a computer program, the supportive and conversational tone of the messages has the potential to lead to a long-term social-emotional connection. Indeed, the interim trial result of a mean of 72 days from first to last use is longer than we have observed in rule-based SMS text messaging interventions for smoking cessation (which typically last about 7 days) [85,99,100] and longer than typical human clinician–delivered interventions, such as telephone quit coaching (which typically last about 7 days) [101,102]. The length of intervention engagement is a strong predictor of treatment success [103,104], so these initial results on QuitBot’s engagement certainly appear promising.

While we developed and tested QuitBot in the United States for an English-speaking audience, the program could be tailored to other nationalities and languages across the world. As a health behavioral change platform, QuitBot has the potential to be adapted to other behavior changes, including alcohol and drug use, dietary change, and physical activity.

### Lessons Learned

QuitBot was developed in the midst of rapid changes in LLM technology, during what is arguably one of the most rapidly transformative periods of AI history (2020 to 2023) [105,106]. Thus, the most important lesson we learned was the value of investing the time in continuing to iterate and improve on our free-form QnA feature as new LLMs were continuously being released. Indeed, when we started to develop the free-form QnA feature in 2020, LLM capabilities were primitive by the current (January 2024) standards. Despite being based on 345 *million* parameters, we learned that DialoGPT was limited in its ability to determine the intent of our questions. By the time GPT-3.5 was released (based on 175 *billion* parameters), the performance of the free-form QnA feature was far superior, which in turn allowed us to improve the quality of answers provided by Azure QnA.

The second most important lesson we learned is the challenge of training an LLM model for a specific clinical domain. The popular press has provided ample examples of LLMs such as GPT providing very detailed answers to questions in a wide variety of topics [105,107,108]. While much has been written about the tendency for LLMs to “hallucinate” (ie, providing confident-sounding answers that are factually wrong or fabricated) [109], the more common problem we encountered in our development process was providing an extensive knowledge base to address highly specific questions within a clinical domain. In our experience, off-the-shelf LLMs are like dilettantes: they possess broad knowledge but lack depth in a particular subject. From this project, we glean that this characteristic holds particularly true when the subject matter requires clinical expertise and familiarity with scientific literature within a specific clinical domain. Overcoming this challenge required multiple iterations to build a knowledge base grounded in empirically supported best practices for smoking cessation. The responses needed to be accurate and clinically sensitive, suggesting that a similar knowledge-building process will be essential for developing chatbots in any other clinical domain.

### Conclusions

The development process yielded a comprehensive, fully developed, quit smoking program delivered through a conversational chatbot. Iterative testing led to improvements in the delivery platform, and a core LLM–supported conversational feature was integrated, enabling users to pose open-ended questions about quitting cigarette smoking. Our next step is testing QuitBot’s efficacy for smoking cessation in a full-scale RCT.

## Appendix

Multimedia Appendix 1 Representative screenshots of QuitBot.

Multimedia Appendix 2 CONSORT eHealth checklist.

## Abbreviations

AI: artificial intelligence
FDA: Food and Drug Administration
FM: Facebook Messenger
LLM: large language model
NCI: National Cancer Institute
OR: odds ratio
PPA: point prevalence abstinence
RCT: randomized controlled trial
SFT: SmokefreeTXT
UX: user experience

## References

[1] Jha P (2009). Avoidable global cancer deaths and total deaths from smoking. Nat Rev Cancer.

[2] Safiri S, Nejadghaderi SA, Abdollahi M, Carson-Chahhoud K, Kaufman JS, Bragazzi NL, Moradi-Lakeh M, Mansournia MA, Sullman MJ, Almasi-Hashiani A, Taghizadieh A, Collins GS, Kolahi AA (2022). Global, regional, and national burden of cancers attributable to tobacco smoking in 204 countries and territories, 1990-2019. Cancer Med.

[3] (2020). Smoking cessation: a report of the surgeon general. U.S. Department of Health and Human Services, Centers for Disease Control and Prevention, National Center for Chronic Disease Prevention and Health Promotion, Office on Smoking and Health.

[4] Patnode CD, Henderson JT, Coppola EL, Melnikow J, Durbin S, Thomas RG (2021). Interventions for tobacco cessation in adults, including pregnant persons: updated evidence report and systematic review for the US preventive services task force. JAMA.

[5] Littman D, Sherman SE, Troxel AB, Stevens ER (2022). Behavioral economics and tobacco control: current practices and future opportunities. Int J Environ Res Public Health.

[6] Choi K, Jones JT, Ruybal AL, McNeel TS, Duarte DA, Webb Hooper MW (2023). Trends in education-related smoking disparities among U.S. Black or African American and White adults: intersections of race, sex, and region. Nicotine Tob Res.

[7] Ho JY, Elo IT (2013). The contribution of smoking to black-white differences in U.S. mortality. Demography.

[8] Kcomt L, Evans-Polce RJ, Engstrom CW, West BT, McCabe SE (2021). Discrimination, sexual orientation discrimination, and severity of tobacco use disorder in the United States: results from the national epidemiologic survey on alcohol and related conditions-III. Nicotine Tob Res.

[9] Arrazola RA, Griffin T, Lunsford NB, Kittner D, Bammeke P, Courtney-Long EA, Armour BS (2023). US cigarette smoking disparities by race and ethnicity - keep going and going!. Prev Chronic Dis.

[10] Girvalaki C, Mechili E, Papadakis S, Nikitara K, Demin A, Trofor A, Lila A, Harutyunyan A, Saliaj A, Dimitrievska D, Lozano FR, Bakh-Turidze G, Ayesta J, Przewozniak K, Cattaruzza M, Zdraveska M, Lovše M, Kilibarda B, Stoyka O, Behrakis P, Bizel P, Starchenko P, Spahija S, Radu-Loghin C, Vardavas C (2020). Current practices and perceived barriers to tobacco-treatment delivery among healthcare professionals from 15 European countries. The EPACTT Plus project. Tob Prev Cessat.

[11] Sakka S, Al-Shatanawi TN, Bataineh DZ, Haddad W, Al Tamimi S, AL Salamat H, Al-Mistarihi AH, Alsulaiman J, Kheirallah K (2022). Knowledge, attitude, practice and perceived barriers towards smoking cessation services among community pharmacists. Pharm Pract.

[12] Julius RJ, Novitsky MA Jr, Dubin WR (2009). Medication adherence: a review of the literature and implications for clinical practice. J Psychiatr Pract.

[13] Twyman L, Bonevski B, Paul C, Bryant J (2014). Perceived barriers to smoking cessation in selected vulnerable groups: a systematic review of the qualitative and quantitative literature. BMJ Open.

[14] Pipe AL, Evans W, Papadakis S (2022). Smoking cessation: health system challenges and opportunities. Tob Control.

[15] Smoking cessation—the role of healthcare professionals and health systems. Centers for Disease Control and Prevention.

[16] (2023). WHO report on the global tobacco epidemic, 2023: protect people from tobacco smoke. World Health Organization.

[17] Babb S, Malarcher A, Schauer G, Asman K, Jamal A (2017). Quitting smoking among adults - United States, 2000-2015. MMWR Morb Mortal Wkly Rep.

[18] Horvath AO, Greenberg LS (1994). The Working Alliance: Theory, Research, and Practice.

[19] Meier PS, Barrowclough C, Donmall MC (2005). The role of the therapeutic alliance in the treatment of substance misuse: a critical review of the literature. Addiction.

[20] Becker MH, Rosenstock IM, Steptoe A, Matthews A (1984). Compliance with medical advice. Health Care and Human Behavior.

[21] Boardman T, Catley D, Grobe JE, Little TD, Ahluwalia JS (2006). Using motivational interviewing with smokers: do therapist behaviors relate to engagement and therapeutic alliance?. J Subst Abuse Treat.

[22] Joe GW, Simpson DD, Dansereau DF, Rowan-Szal GA (2001). Relationships between counseling rapport and drug abuse treatment outcomes. Psychiatr Serv.

[23] Gardiner PM, McCue KD, Negash LM, Cheng T, White LF, Yinusa-Nyahkoon L, Jack BW, Bickmore TW (2017). Engaging women with an embodied conversational agent to deliver mindfulness and lifestyle recommendations: a feasibility randomized control trial. Patient Educ Couns.

[24] Provoost S, Lau HM, Ruwaard J, Riper H (2017). Embodied conversational agents in clinical psychology: a scoping review. J Med Internet Res.

[25] Bickmore TW, Utami D, Matsuyama R, Paasche-Orlow MK (2016). Improving access to online health information with conversational agents: a randomized controlled experiment. J Med Internet Res.

[26] King AC, Campero I, Sheats JL, Castro Sweet CM, Garcia D, Chazaro A, Blanco G, Hauser M, Fierros F, Ahn DK, Diaz J, Done M, Fernandez J, Bickmore T (2017). Testing the comparative effects of physical activity advice by humans vs. computers in underserved populations: the COMPASS trial design, methods, and baseline characteristics. Contemp Clin Trials.

[27] Ayers JW, Poliak A, Dredze M, Leas EC, Zhu Z, Kelley JB, Faix DJ, Goodman AM, Longhurst CA, Hogarth M, Smith DM (2023). Comparing physician and artificial intelligence chatbot responses to patient questions posted to a public social media forum. JAMA Intern Med.

[28] Whittaker R, Dobson R, Garner K (2022). Chatbots for smoking cessation: scoping review. J Med Internet Res.

[29] Bendotti H, Lawler S, Chan GC, Gartner C, Ireland D, Marshall HM (2023). Conversational artificial intelligence interventions to support smoking cessation: a systematic review and meta-analysis. Digit Health.

[30] (2020). AI for quitting tobacco initiative. World Health Organization.

[31] Quit with Bella homepage. Quit With Bella.

[32] Alex - quit smoking. Alex Therapeutics.

[33] Loveys K, Antoni M, Donkin L, Sagar M, Broadbent E (2023). Comparing the feasibility and acceptability of a virtual human, teletherapy, and an e-manual in delivering a stress management intervention to distressed adult women: pilot study. JMIR Form Res.

[34] de Vito Dabbs A, Myers B, Mc Curry KR, Dunbar-Jacob J, Hawkins R, Begey A, Dew MA (2009). User-centered design and interactive health technologies for patients. Comput Inform Nurs.

[35] Bendotti H, Ireland D, Lawler S, Oates D, Gartner C, Marshall HM (2024). Introducing quin: the design and development of a prototype chatbot to support smoking cessation. Nicotine Tob Res.

[36] Almusharraf F, Rose J, Selby P (2020). Engaging unmotivated smokers to move toward quitting: design of motivational interviewing-based chatbot through iterative interactions. J Med Internet Res.

[37] (2019). ISO 9241-210:2019 Ergonomics of human-system interaction: part 210: human-centred design for interactive systems. International Organization for Standardization.

[38] ISO 13407:1999(en) Human-centred design processes for interactive systems. International Organization for Standardization.

[39] Warlick C, Richter KP, Catley D, Gajewski BJ, Martin LE, Mussulman LM (2018). Two brief valid measures of therapeutic alliance in counseling for tobacco dependence. J Subst Abuse Treat.

[40] Klemperer EM, Hughes JR, Callas PW, Solomon LJ (2017). Working alliance and empathy as mediators of brief telephone counseling for cigarette smokers who are not ready to quit. Psychol Addict Behav.

[41] Havens L (1986). Making Contact: Uses of Language in Psychotherapy.

[42] Okun BF, Kantrowitz RE (1976). Effective Helping: Interviewing and Counseling Techniques.

[43] Laver J (1975). Communicative functions of phatic communion. Organization of Behavior in Face-to-Face Interaction.

[44] Dainton M, Stafford L (1993). Routine maintenance behaviors: a comparison of relationship type, partner similarity and sex differences. J Soc Pers Relatsh.

[45] Stafford L, Canary DJ (1991). Maintenance strategies and romantic relationship type, gender and relational characteristics. J Soc Pers Relatsh.

[46] Brown P, Levinson SC (1987). Politeness: Some Universals in Language Usage.

[47] Laver J (1981). Linguistic routines and politeness in greeting and parting. Conversational Routine.

[48] Bickmore T, Gruber A, Picard R (2005). Establishing the computer-patient working alliance in automated health behavior change interventions. Patient Educ Couns.

[49] Archer RL, Wegner DM, Vallacher RR (1980). Self-disclosure. The Self in Social Psychology.

[50] Shenk CE, Fruzzetti AE (2011). The impact of validating and invalidating responses on emotional reactivity. J Soc Clin Psychol.

[51] Ho A, Hancock J, Miner AS (2018). Psychological, relational, and emotional effects of self-disclosure after conversations with a chatbot. J Commun.

[52] Clinical Practice Guideline Treating Tobacco Use and Dependence 2008 Update Panel, Liaisons, and Staff (2008). A clinical practice guideline for treating tobacco use and dependence: 2008 update. A U.S. Public Health Service report. Am J Prev Med.

[53] Bricker JB, Sullivan BM, Mull KE, Torres AJ, Carpenter KM (2022). Full-scale randomized trial comparing acceptance and commitment therapy telephone-delivered coaching with standard telephone-delivered coaching among Medicare/uninsured Quitline callers. Nicotine Tob Res.

[54] (2024). What is language understanding (LUIS)?. Microsoft.

[55] Bricker JB, Mull KE, Santiago-Torres M, Miao Z, Perski O, Di C (2022). Smoking cessation smartphone app use over time: predicting 12-month cessation outcomes in a 2-arm randomized trial. J Med Internet Res.

[56] Sharp H, Preece J, Rogers Y (2019). Interaction Design: Beyond Human-Computer Interaction.

[57] (2011). Human Centered Design Toolkit, Second Edition.

[58] Braun V, Clarke V (2006). Using thematic analysis in psychology. Qual Res Psychol.

[59] Botmock. crunchbase.

[60] Botmock. Wayback Machine.

[61] Chatfuel homepage. Chatfuel.

[62] Fiore M (2008). Treating Tobacco Use and Dependence: 2008 Update.

[63] Kraus R (2018). Facebook is really proud of its 300,000 business bots, despite claiming it will put ’people first’. Mashable.

[64] Number of Facebook Messenger users in the United States from 2018 to 2022. Statista.

[65] Acceptance of artificial intelligence chatbots by customers worldwide, as of 2017, by service. Statista.

[66] Node.js® - run JavaScript everywhere. OpenJS Foundation.

[67] Guest G, Bunce A, Johnson L (2006). How many interviews are enough?: an experiment with data saturation and variability. Field Methods.

[68] Daae J, Boks C (2015). A classification of user research methods for design for sustainable behaviour. J Clean Prod.

[69] Portz JD, Bayliss EA, Bull S, Boxer RS, Bekelman DB, Gleason K, Czaja S (2019). Using the technology acceptance model to explore user experience, intent to use, and use behavior of a patient portal among older adults with multiple chronic conditions: descriptive qualitative study. J Med Internet Res.

[70] Leon AC, Davis LL, Kraemer HC (2011). The role and interpretation of pilot studies in clinical research. J Psychiatr Res.

[71] Rounsaville BJ, Carroll KM, Onken LS (2001). A stage model of behavioral therapies research: getting started and moving on from stage I. Clin Psychol Sci Pract.

[72] Bricker J, Wyszynski C, Comstock B, Heffner JL (2013). Pilot randomized controlled trial of web-based acceptance and commitment therapy for smoking cessation. Nicotine Tob Res.

[73] Bricker JB, Mull KE, Kientz JA, Vilardaga R, Mercer LD, Akioka KJ, Heffner JL (2014). Randomized, controlled pilot trial of a smartphone app for smoking cessation using acceptance and commitment therapy. Drug Alcohol Depend.

[74] Boland VC, Stockings EA, Mattick RP, McRobbie H, Brown J, Courtney RJ (2018). The methodological quality and effectiveness of technology-based smoking cessation interventions for disadvantaged groups: a systematic review and meta-analysis. Nicotine Tob Res.

[75] Chen YF, Madan J, Welton N, Yahaya I, Aveyard P, Bauld L, Wang D, Fry-Smith A, Munafò MR (2012). Effectiveness and cost-effectiveness of computer and other electronic aids for smoking cessation: a systematic review and network meta-analysis. Health Technol Assess.

[76] Graham AL, Amato MS (2019). Twelve million smokers look online for smoking cessation help annually: health information national trends survey data, 2005-2017. Nicotine Tob Res.

[77] Hall AK, Cole-Lewis H, Bernhardt JM (2015). Mobile text messaging for health: a systematic review of reviews. Annu Rev Public Health.

[78] Rodgers A, Corbett T, Bramley D, Riddell T, Wills M, Lin RB, Jones M (2005). Do u smoke after txt? results of a randomised trial of smoking cessation using mobile phone text messaging. Tob Control.

[79] Cheung YT, Chan CH, Lai CK, Chan WF, Wang MP, Li HC, Chan SS, Lam TH (2015). Using WhatsApp and Facebook online social groups for smoking relapse prevention for recent quitters: a pilot pragmatic cluster randomized controlled trial. J Med Internet Res.

[80] Durmaz S, Ergin I, Durusoy R, Hassoy H, Caliskan A, Okyay P (2019). WhatsApp embedded in routine service delivery for smoking cessation: effects on abstinence rates in a randomized controlled study. BMC Public Health.

[81] ICF International homepage. ICF International.

[82] Heinzen E, Sinnwell J, Atkinson E, Gunderson T, Dougherty G, Votruba P, Lennon R, Hanson A, Goergen K, Lundt E, Broderick B, McCullough M (2021). arsenal: an arsenal of 'R' functions for large-scale statistical summaries. The Comprehensive R Archive Network.

[83] R Core Team (2019). R: a language and environment for statistical computing. R Foundation for Statistical Computing.

[84] Venables WN, Ripley BD (2002). Modern Applied Statistics with S.

[85] Whittaker R, McRobbie H, Bullen C, Rodgers A, Gu Y (2016). Mobile phone-based interventions for smoking cessation. Cochrane Database Syst Rev.

[86] Holm S (1979). A simple sequentially rejective multiple test procedure. Scand J Stat.

[87] Watson NL, Mull KE, Heffner JL, McClure JB, Bricker JB (2018). Participant recruitment and retention in remote eHealth intervention trials: methods and lessons learned from a large randomized controlled trial of two web-based smoking interventions. J Med Internet Res.

[88] Scott-Sheldon LA, Lantini R, Jennings EG, Thind H, Rosen RK, Salmoirago-Blotcher E, Bock BC (2016). Text messaging-based interventions for smoking cessation: a systematic review and meta-analysis. JMIR Mhealth Uhealth.

[89] Taylor GM, Dalili MN, Semwal M, Civljak M, Sheikh A, Car J (2017). Internet-based interventions for smoking cessation. Cochrane Database Syst Rev.

[90] Counseling and psychotherapy transcripts: volume I. Alexander Street.

[91] Get live, online assistance from NCI's Cancer Information Service. National Institutes of Health National Cancer Institute.

[92] Bricker JB, Mull KE, McClure JB, Watson NL, Heffner JL (2018). Improving quit rates of web-delivered interventions for smoking cessation: full-scale randomized trial of WebQuit.org versus Smokefree.gov. Addiction.

[93] Bricker JB, Watson NL, Mull KE, Sullivan BM, Heffner JL (2020). Efficacy of smartphone applications for smoking cessation: a randomized clinical trial. JAMA Intern Med.

[94] Models. OpenAI Platform.

[95] Wang J (2019). Formal Methods in Computer Science.

[96] Alphonse A, Stewart K, Brown J, Perski O (2022). Exploring users' experiences with a quick-response chatbot within a popular smoking cessation smartphone app: semistructured interview study. JMIR Form Res.

[97] Abdullah AS, Gaehde S, Bickmore T (2018). A tablet based embodied conversational agent to promote smoking cessation among veterans: a feasibility study. J Epidemiol Glob Health.

[98] Olano-Espinosa E, Avila-Tomas JF, Minue-Lorenzo C, Matilla-Pardo B, Serrano Serrano ME, Martinez-Suberviola FJ, Gil-Conesa M, Del Cura-González I (2022). Effectiveness of a conversational chatbot (Dejal@bot) for the adult population to quit smoking: pragmatic, multicenter, controlled, randomized clinical trial in primary care. JMIR Mhealth Uhealth.

[99] Christofferson DE, Hertzberg JS, Beckham JC, Dennis PA, Hamlett-Berry K (2016). Engagement and abstinence among users of a smoking cessation text message program for veterans. Addict Behav.

[100] Coa KI, Wiseman KP, Higgins B, Augustson E (2019). Associations between engagement and outcomes in the SmokefreeTXT program: a growth mixture modeling analysis. Nicotine Tob Res.

[101] Piñeiro B, Wetter DW, Vidrine DJ, Hoover DS, Frank-Pearce SG, Nguyen N, Zbikowski SM, Williams MB, Vidrine JI (2019). Quitline treatment dose predicts cessation outcomes among safety net patients linked with treatment via Ask-Advise-Connect. Prev Med Rep.

[102] Matkin W, Ordóñez-Mena JM, Hartmann-Boyce J (2019). Telephone counselling for smoking cessation. Cochrane Database Syst Rev.

[103] Heminger CL, Boal AL, Zumer M, Abroms LC (2016). Text2Quit: an analysis of participant engagement in the mobile smoking cessation program. Am J Drug Alcohol Abuse.

[104] Zeng EY, Heffner JL, Copeland WK, Mull KE, Bricker JB (2016). Get with the program: adherence to a smartphone app for smoking cessation. Addict Behav.

[105] Toloka Team (2023). The history, timeline, and future of LLMs. Toloka.

[106] Tucci L (2024). A guide to artificial intelligence in the enterprise. TechTarget.

[107] Bushwick S (2023). What the new GPT-4 AI can do. Scientific American.

[108] Roose K (2023). GPT-4 is exciting and scary. The New York Times.

[109] Metz C, Collins K (2023). 10 ways GPT-4 is impressive but still flawed. The New York Times.

[110] How can large language models help combat addiction | The Prompt with Trevor Noah. YouTube.

