# Supplementary figures and images for: Conversational Chatbot for Cigarette Smoking Cessation: Results From the 11-Step User-Centered Design Development Process and Randomized Controlled Trial
Source: JMIR Mhealth Uhealth. 2024 Jul 23;12:e57318. doi: 10.2196/57318 (PMC11303891; doi:10.2196/57318)

Representative screenshots of QuitBot


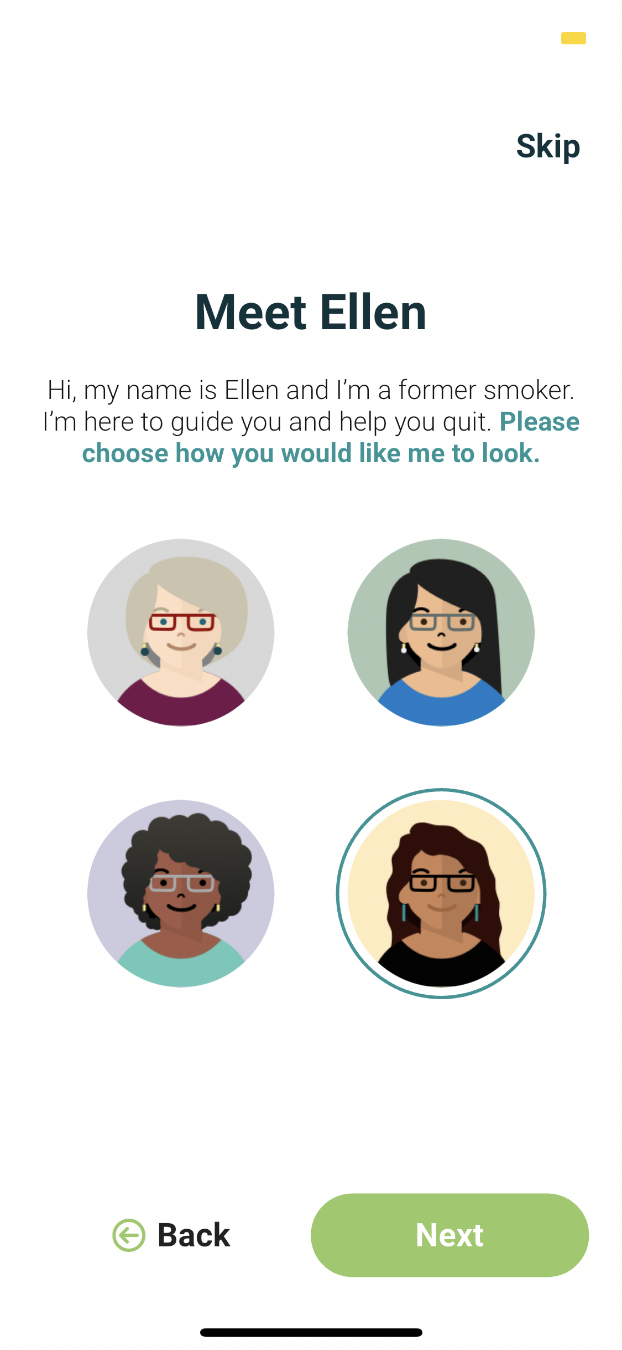

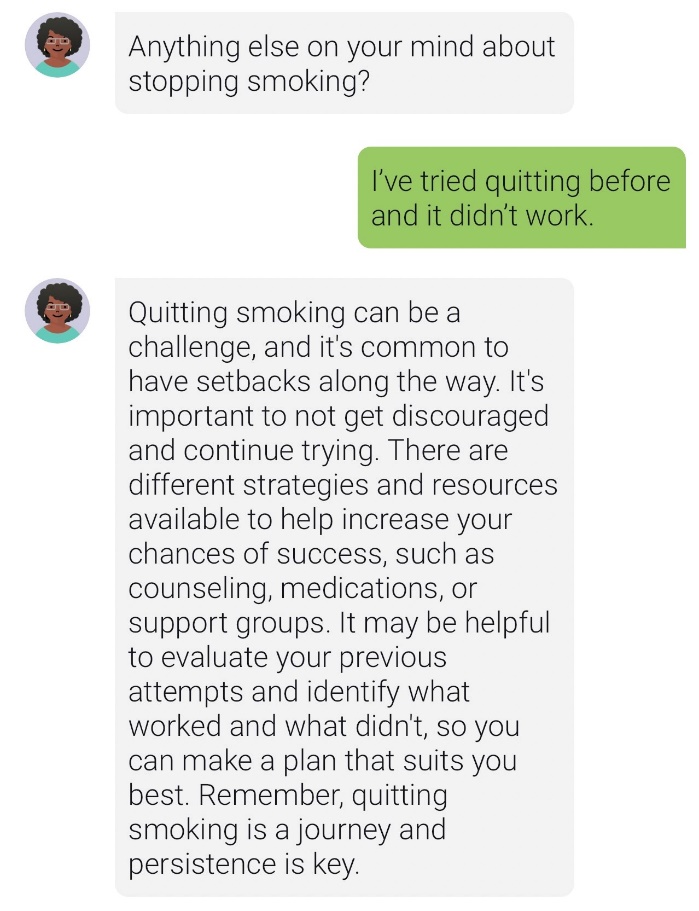


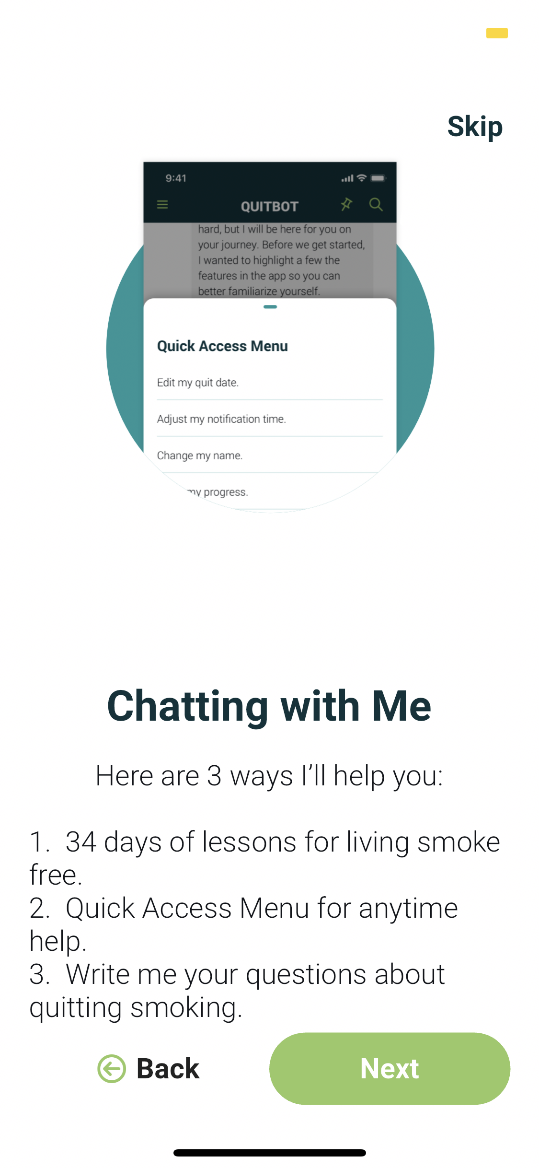

Supplement: Multimedia Appendix 1 [file mhealth_v12i1e57318_app1.docx]
