# Supplementary material for: Conversational Chatbot for Cigarette Smoking Cessation: Results From the 11-Step User-Centered Design Development Process and Randomized Controlled Trial
Source: JMIR Mhealth Uhealth. 2024 Jul 23;12:e57318. doi: 10.2196/57318 (PMC11303891; doi:10.2196/57318)
Supplement: Multimedia Appendix 2 [file mhealth_v12i1e57318_app2.pdf]

CONSORT-EHEALTH Checklist V1.6.2 Report	Manuscript Number	57318
**(based on CONSORT-EHEALTH V1.6), available at [http://tinyurl.com/consort-ehealth-v1-6].**		
**Date completed**		
2/15/2024 19:23:30		
**by**		
Jonathan Bricker		
Development of a Large Language Model-Supported Conversational Chatbot for Cigarette Smoking Cessation		
TITLE		
**1a-i) Identify the mode of delivery in the title**		
"Conversational Chatbot" is in the title		
**1a-ii) Non-web-based components or important co-interventions in title**		
**1a-iii) Primary condition or target group in the title**		
Cigarette Smoking Cessation		
ABSTRACT		
**1b-i) Key features/functionalities/components of the intervention and comparator in the METHODS section of the ABSTRACT**		
The four years of formative research for developing QuitBot followed an eleven-step process: (1) specifying a conceptual model, (2) conducting content analysis of existing interventions, (3) assessing user needs, (4) developing the chat's persona ("personality"), (5) prototyping content and persona, (6) developing full functionality, (7) programming the QuitBot, (8) conducting a diary study, (9) conducting a pilot randomized trial, (10) reviewing results of the trial, and (11) adding a free-form question and answer (QnA) function, based on user feedback from pilot trial results. The process of adding a QnA function itself involved a three-step process: (a) generating QnA pairs, (b) fine tuning Large Language Models (LLMs) on QnA pairs, and (c) evaluating the LLM model outputs.		
**1b-ii) Level of human involvement in the METHODS section of the ABSTRACT**		
The four years of formative research for developing QuitBot followed an eleven-step process: (1) specifying a conceptual model, (2) conducting content analysis of existing interventions, (3) assessing user needs, (4) developing the chat's persona ("personality"), (5) prototyping content and persona, (6) developing full functionality, (7) programming the QuitBot, (8) conducting a diary study, (9) conducting a pilot randomized trial, (10) reviewing results of the trial, and (11) adding a free-form question and answer (QnA) function, based on user feedback from pilot trial results. The process of adding a QnA function itself involved a three-step process: (a) generating QnA pairs, (b) fine tuning Large Language Models (LLMs) on QnA pairs, and (c) evaluating the LLM model outputs.		
**1b-iii) Open vs. closed, web-based (self-assessment) vs. face-to-face assessments in the METHODS section of the ABSTRACT**		
Focus of the abstract is not on blinding of the trial.		
**1b-iv) RESULTS section in abstract must contain use data**		
A quit smoking program spanning 42 days of 2 to 3-minute conversations over two phases: (1) pre-quitting phase (14 days) content covering topics ranging from motivations to quit, setting a quit date, choosing FDA-approved cessation medications; and (2) post-quit phase (28 days) content on coping with triggers and recovering from lapses/relapses. In a pilot randomized trial with 96% three-month outcome data retention, QuitBot demonstrated high user engagement and promising cessation rates compared to the National Cancer Institute's SmokefreeTXT (SFT) text messaging program—particularly among those who viewed all 42 days of program content: 30-day complete-case, point prevalence abstinence (PPA) rates at three-month follow-up were 63% (39/62) for QuitBot vs. 38% (45/117) for SFT (OR = 2.58; 95% CI: 1.34, 4.99; P =.005). However, Facebook Messenger (FM) intermittently blocked participants' access to QuitBot so we transitioned from FM to a standalone smartphone app as the communication channel. Participants' frustration with QuitBot's inability to answer their open-ended questions lead to us develop a core conversational feature enabling users to ask open-ended questions about quitting cigarette smoking and for the QuitBot to respond with accurate and professional answers. To support this functionality, we developed a library of 11,000 QnA pairs on topics associated with quitting cigarette smoking. Model testing results showed that Microsoft's Azure-based QnA maker effectively handled questions that matched our library of 11,000 QnA pairs. A fine-tuned, contextualized GPT3.5 responds to questions that are not within our library of QnA pairs.		
**1b-v) CONCLUSIONS/DISCUSSION in abstract for negative trials**		
Not a negative trial being reported		

INTRODUCTION		
**2a-i) Problem and the type of system/solution**		
Cigarette smoking accounts for 8 million premature deaths and 25% of all cancer deaths annually [1, 2]. Despite advancements in government policies, anti-smoking campaigns, and shifting societal norms, existing smoking cessation interventions continue to have limited treatment engagement and cessation rates [3-9]. While this is a problem for the general population of people who smoke, the issue is particularly pronounced in marginalized communities, synonymous with vulnerable or disadvantaged groups, which are segments of society facing systemic disadvantages and barriers in accessing resources and opportunities. Marginalized populations, marked by factors such as racial or ethnic minority status, sexual or gender identity differences, low education and income levels, higher unemployment rates, and/or an increased prevalence of mental illness, encounter discrimination, social exclusion, and limited influence in decision-making processes.		
**2a-ii) Scientific background, rationale: What is known about the (type of) system**		
A review of existing literature yielded a limited number of empirical studies, and those available often exhibit low methodological quality [28]. There is a notable paucity of randomized controlled trials (RCTs) focusing on conversational chatbots for smoking cessation, and while promising results have emerged, they have been limited by low quit rates [29]. Several conversational chatbots for smoking cessation in the public domain include Florence [30], Bella [31], and Alex AI [32]. However, we are not aware of publications on their efficacy, with only the Florence app having reported user's receptivity results [33]. Critical to creating useful and engaging conversational chatbots is following a user-centered design development process [34]. Like most chatbots, the development of the chatbots listed above has followed a "top-down" approach, lacking a user-centered design that involved conducting a needs assessment or including user feedback during the development process [28, 35].		
**Does your paper address CONSORT subitem 2b?**		
To address this gap, this paper aims to describe the comprehensive four-year, eleven-step user-centered design development process for a novel quit smoking conversational chatbot named "QuitBot".		
METHODS		
**3a) CONSORT: Description of trial design (such as parallel, factorial) including allocation ratio**		
The favorable feedback from the diary study led us to conduct a three-arm pilot RCT comparing QuitBot (n = 200) to the Smokefree TXT (n = 149) intervention and to a QuitBot delayed access control group (n = 55).		
**3b) CONSORT: Important changes to methods after trial commencement (such as eligibility criteria), with reasons**		
No changes were made after commencement		
**3b-i) Bug fixes, Downtimes, Content Changes**		
There were no bug fixes, downtimes, or content changes.		
**4a) CONSORT: Eligibility criteria for participants**		
Eligibility. (1) age 18 and older, (2) having smoked at least one cigarette a day for at least the past 12 months, (3) wanting to quit cigarette smoking within the next 14 days, (4) if concurrently using any other nicotine or tobacco products, wanting to quit using them within the next 14 days, (5) being interested in learning skills to quit smoking, (6) being willing to be randomly assigned to either condition, (7) residing in the US, (8) having daily access to their own smartphone, (9) having both text messaging and FM on their smartphone (criterion 8 and 9 were required to receive each interventions' content), (10) being willing and able to read in English, and (11) not using other smoking cessation interventions. Individuals deemed ineligible to participate were directed to the smokefree.gov website and the 800-QUIT-NOW number for access to their state's quitline resources.		
**4a-i) Computer / Internet literacy**		
Intervention designed for broad reach, a wide range of computer literacy		
**4a-ii) Open vs. closed, web-based vs. face-to-face assessments:**		
Facebook ads deployed across the US		
**4a-iii) Information giving during recruitment**		
Online recruitment survey and informed consent form.		
**4b) CONSORT: Settings and locations where the data were collected**		
Facebook ads recruited participants from the around the US		
**4b-i) Report if outcomes were (self-)assessed through online questionnaires**		
30-day cessation at the three month outcome		
**4b-ii) Report how institutional affiliations are displayed**		
Fred Hutch Cancer Center		
**5) CONSORT: Describe the interventions for each group with sufficient details to allow replication, including how and when they were actually administered**		

**5-i) Mention names, credential, affiliations of the developers, sponsors, and owners**		
This is a free and publicly available intervention developed by the authors		
**5-ii) Describe the history/development process**		
Development is the focus of this paper. See Steps 1 to 11 of the paper, which covers the entire Methods section.		
**5-iii) Revisions and updating**		
No changes made during pilot trial (Step 9).		
**5-iv) Quality assurance methods**		
Double blind RCT design was employed		
**5-v) Ensure replicability by publishing the source code, and/or providing screenshots/screen-capture video, and/or providing flowcharts of the algorithms used**		
QuitBot is freely available at QuitBot.net.		
**5-vi) Digital preservation**		
QuitBot is freely available at QuitBot.net		
**5-vii) Access**		
QuitBot is freely available at QuitBot.net		
**5-viii) Mode of delivery, features/functionalities/components of the intervention and comparator, and the theoretical framework**		
Final Version of QuitBot<br>The final version of QuitBot is a standalone app that features (a) a personal coach (named “Ellen”) who supports the user, (b) a series of 42 days of 2 to 3-minute structured clinical conversations with Ellen, guiding the user through distinct stages of quitting smoking and (c) the ability for users to pose any freeform question related to quitting smoking. The structured conversations provide the valuable function of a clear step-by-step program for staying motivated, learning about one’s triggers to smoke, setting a quit date, and staying smoke-free. Complementing the structured conversations, the freeform question feature provides users the freedom to ask their own questions, the option to address unique clinical needs, and the opportunity to follow-up on the content provided in the structured conversations. The combination of both structured and freeform conversation features is intended to balance their main strengths and limitations: the structured clinical format offers a guided program on quitting smoking, albeit with limited user question flexibility, while the open-ended format provides freedom but may encounter instances of not fully understanding the user’s questions to give them clear guidance—despite the positive performance of the QnA feature thus far.		
**5-ix) Describe use parameters**		
used the QuitBot ad libitum		
**5-x) Clarify the level of human involvement**		
No human involvement in the intervention delivery		
**5-xi) Report any prompts/reminders used**		
No prompts to use the application were provided by the research team or any of the trial operations.		
**5-xii) Describe any co-interventions (incl. training/support)**		
No co-interventions were provided		
**6a) CONSORT: Completely defined pre-specified primary and secondary outcome measures, including how and when they were assessed**		
The number of times participants interacted with their assigned intervention		
**6a-i) Online questionnaires: describe if they were validated for online use and apply CHERRIES items to describe how the questionnaires were designed/deployed**		
Number of times interacted with assigned intervention was objective data collected from the server, not self-reported.		
**6a-ii) Describe whether and how “use” (including intensity of use/dosage) was defined/measured/monitored**		
Number of times interacted with assigned intervention was objective data collected from the server,		
**6a-iii) Describe whether, how, and when qualitative feedback from participants was obtained**		
Interviews and feedback forms		
**6b) CONSORT: Any changes to trial outcomes after the trial commenced, with reasons**		
Facebook ads recruited participants from the around the US		
**7a) CONSORT: How sample size was determined**		
**7a-i) Describe whether and how expected attrition was taken into account when calculating the sample size**		

Pilot exploratory trial so no prior data or estimate effect size drove the sample size.		
**7b) CONSORT: When applicable, explanation of any interim analyses and stopping guidelines**		
The number of times participants interacted with their assigned intervention		
**8a) CONSORT: Method used to generate the random allocation sequence**		
Based on evidence from text messaging trials meta-analyses [71], we stratified randomization on biological sex (male vs. female), heaviness of smoking index score (≤ 4 vs >4), and percent confidence in being smoke-free in 12 months (≤ 70% vs. >70%).		
**8b) CONSORT: Type of randomisation; details of any restriction (such as blocking and block size)**		
Based on evidence from text messaging trials meta-analyses [71], we stratified randomization on biological sex (male vs. female), heaviness of smoking index score (≤ 4 vs >4), and percent confidence in being smoke-free in 12 months (≤ 70% vs. >70%).		
**9) CONSORT: Mechanism used to implement the random allocation sequence (such as sequentially numbered containers), describing any steps taken to conceal the sequence until interventions were assigned**		
Sequential allocation based on cell sizes		
**10) CONSORT: Who generated the random allocation sequence, who enrolled participants, and who assigned participants to interventions**		
Study operations staff blinded to participants		
**11a) CONSORT: Blinding - If done, who was blinded after assignment to interventions (for example, participants, care providers, those assessing outcomes) and how**		
**11a-i) Specify who was blinded, and who wasn't**		
Double blinded to the entire research team and trial participants		
**11a-ii) Discuss e.g., whether participants knew which intervention was the "intervention of interest" and which one was the "comparator"**		
Double blinded. For blinding, both interventions were called "QuitBot."		
**11b) CONSORT: If relevant, description of the similarity of interventions**		
See Table 1 for this information		
**12a) CONSORT: Statistical methods used to compare groups for primary and secondary outcomes**		
Does not include as not focus of the paper. Statistics reported were standard.		
**12a-i) Imputation techniques to deal with attrition / missing values**		
Missing equals smoking See Table 2		
**12b) CONSORT: Methods for additional analyses, such as subgroup analyses and adjusted analyses**		
For those who completed their assigned intervention (i.e., viewed all 42 days of planned content)		
RESULTS		
**13a) CONSORT: For each group, the numbers of participants who were randomly assigned, received intended treatment, and were analysed for the primary outcome**		
These efforts yielded 2954 participants screened, 1380 eligible, 583 consented, and 418 randomized. After completion of study participation, 14 participants were found to be cases of fraud, duplicate participants, or in the same household as another participant, leading to a total of 404 participants included in analyses.		
**13b) CONSORT: For each group, losses and exclusions after randomisation, together with reasons**		
These efforts yielded 2954 participants screened, 1380 eligible, 583 consented, and 418 randomized. After completion of study participation, 14 participants were found to be cases of fraud, duplicate participants, or in the same household as another participant, leading to a total of 404 participants included in analyses.		
**13b-i) Attrition diagram**		
See Table 2 for number of engagements and first to last engagement		
**14a) CONSORT: Dates defining the periods of recruitment and follow-up**		
9/17/2018 to 12/6/2019 were the dates of recruitment to follow-up		
**14a-i) Indicate if critical "secular events" fell into the study period**		
No secular events occured		
**14b) CONSORT: Why the trial ended or was stopped (early)**		
Trial stopped when target enrollment obtained		
**15) CONSORT: A table showing baseline demographic and clinical characteristics for each group**		

Participants were on average, 36 years old, 70% female, 29% reported racial or ethnic minority background, 53% were unemployed, 45% had high school or less education, 72% smoked at least half pack daily, and 60% had high cigarette dependence (FTCD scores of 6 or more).		
**15-i) Report demographics associated with digital divide issues**		
Participants were on average, 36 years old, 70% female, 29% reported racial or ethnic minority background, 53% were unemployed, 45% had high school or less education, 72% smoked at least half pack daily, and 60% had high cigarette dependence (FTCD scores of 6 or more).		
**16a) CONSORT: For each group, number of participants (denominator) included in each analysis and whether the analysis was by original assigned groups**		
**16-i) Report multiple "denominators" and provide definitions**		
See Table 2 for this information		
**16-ii) Primary analysis should be intent-to-treat**		
Complete case and missing equals smoking are reported in Table 2		
**17a) CONSORT: For each primary and secondary outcome, results for each group, and the estimated effect size and its precision (such as 95% confidence interval)**		
95% CIs are reported in Table 2		
**17a-i) Presentation of process outcomes such as metrics of use and intensity of use**		
See Table 2 for this information		
**17b) CONSORT: For binary outcomes, presentation of both absolute and relative effect sizes is recommended**		
See Table 2 for this information		
**18) CONSORT: Results of any other analyses performed, including subgroup analyses and adjusted analyses, distinguishing pre-specified from exploratory**		
See Table 2 for this information		
**18-i) Subgroup analysis of comparing only users**		
See Table 2 for this analysis of participants who completed the intervention		
**19) CONSORT: All important harms or unintended effects in each group**		
there were no harms or unintented effects		
**19-i) Include privacy breaches, technical problems**		
No privacy breaches were encountered		
**19-ii) Include qualitative feedback from participants or observations from staff/researchers**		
Comments from QuitBot arm trial participants reflected a strong overall bond with the chatbot's persona: "I loved Ellen. She was always there when I needed her"; "Ellen was always there for me when I had a craving"; "I love how engaged she was, I could really quit with her there to talk to"; "She made me feel like I was not alone"; "She was there without making me feel ashamed"; "She was kind, non-judgmental"; "She held me accountable"; "Felt like a friend encouraging me."<br>Conversely, participants were frustrated by QuitBot's inability to respond to their specific questions about quitting smoking: "I could not ask questions and get real answers back"; "I could not ask it real live questions"; "I wanted to write my own questions"; "Can't ask any question"; "Not being able to respond to my questions"; "I wish you could talk to her…without it being a constant couple of options"; "I didn't like how it selected responses"; "The fact that you cannot ask a question and [it] has no idea what you are saying unless you select one of the options."		
DISCUSSION		
**20) CONSORT: Trial limitations, addressing sources of potential bias, imprecision, multiplicity of analyses**		
**20-i) Typical limitations in ehealth trials**		
QuitBot has several key limitations that might present a challenge for users who expect fast responses to their questions. The QuitBot was designed for users to wait until the end of the 2 to 3-minute structured clinical conversations before they can ask freeform questions. This design element was necessary to prevent breaking the logic of each of the structured conversations, and thereby going off on tangents without an ability to return to the structured conversation. We address this design element by asking the user to hold onto their questions until the end of the structured conversation at various times throughout the program. To date, this message appears to have been effective at training the user to wait until the end of the structured conversation to ask freeform questions. The second major limitation is the freeform question response time latency when the GPT servers are running at capacity. While usually the response latency is only a few seconds, we have observed some instances where it can take up to 30 seconds. We are addressing this potential delay by telling the users that it may take a moment to answer their question, so their patience is appreciated.		
**21) CONSORT: Generalisability (external validity, applicability) of the trial findings**		

**21-i) Generalizability to other populations**		
While we developed and tested QuitBot in the US for an English-speaking audience, the program could be tailored to other nationalities and languages across the world. As a health behavioral change platform, QuitBot has the potential to be adapted to other behavior changes including alcohol and drug use, dietary change, and physical activity.		
**21-ii) Discuss if there were elements in the RCT that would be different in a routine application setting**		
The second major limitation is the freeform question response time latency when the GPT servers are running at capacity. While usually the response latency is only a few seconds, we have observed some instances where it can take up to 30 seconds. We are addressing this potential delay by telling the users that it may take a moment to answer their question, so their patience is appreciated.		
**22) CONSORT: Interpretation consistent with results, balancing benefits and harms, and considering other relevant evidence**		
**22-i) Restate study questions and summarize the answers suggested by the data, starting with primary outcomes and process outcomes (use)**		
This paper described the research group's four-year process of developing a conservational chatbot for cigarette smoking cessation ("QuitBot"). The user-centered development process yielded a comprehensive quit smoking program of 42 days of 2 to 3-minute focused conversations over two phases: a pre-quit phase with 14 days of conversation content and a post-quit phase with 28 days of conversation content. The program content covers topics ranging from motivations to quit, setting a quit date, choosing FDA-approved medications, identifying, and coping with a wide range of triggers to smoke, and recovering from lapses or relapses. The program content, which follows the US Clinical Practice Guidelines for smoking cessation, is presented as a continuous conversation, built on user input from prior conversations. QuitBot is both proactive and on-demand for anytime help. Users can continue to interact with QuitBot after completing the 42 days of conversations.		
**22-ii) Highlight unanswered new questions, suggest future research**		
While we developed and tested QuitBot in the US for an English-speaking audience, the program could be tailored to other nationalities and languages across the world. As a health behavioral change platform, QuitBot has the potential to be adapted to other behavior changes including alcohol and drug use, dietary change, and physical activity.		
Other information		
**23) CONSORT: Registration number and name of trial registry**		
ClinicalTrials.gov Identifier, NCT03585231		
**24) CONSORT: Where the full trial protocol can be accessed, if available**		
Available upon request from the corresponding author		
**25) CONSORT: Sources of funding and other support (such as supply of drugs), role of funders**		
Fred Hutchinson Cancer Research Center		
**X26-i) Comment on ethics committee approval**		
All study procedures were approved by the Fred Hutch Cancer Center Institutional Review Board.		
**x26-ii) Outline informed consent procedures**		
Low risk behavioral intervention so this was not emphasized in the report.		
**X26-iii) Safety and security procedures**		
QuitBot has a link to a suicide crisis line if a participant indicates a crisis in their responses.		
**X27-i) State the relation of the study team towards the system being evaluated**		
None of the authors has a conflict of interest.		
